# Economic and Environmental Performance of an Integrated CO_2_ Refinery

**DOI:** 10.1021/acssuschemeng.2c06724

**Published:** 2023-01-26

**Authors:** Iasonas Ioannou, Juan Javaloyes-Antón, José A. Caballero, Gonzalo Guillén-Gosálbez

**Affiliations:** † Institute for Chemical and Bioengineering, Department of Chemistry and Applied Biosciences, ETH Zürich, Vladimir-Prelog-Weg 1, 8093Zürich, Switzerland; ‡ Institute of Chemical Processes Engineering, University of Alicante, P.O. Box 99, E-03080Alicante, Spain

**Keywords:** carbon capture and utilization (CCU), refinery, Allam cycle, CO_2_ hydrogenation, residual gas utilization (RGU), methanol economy

## Abstract

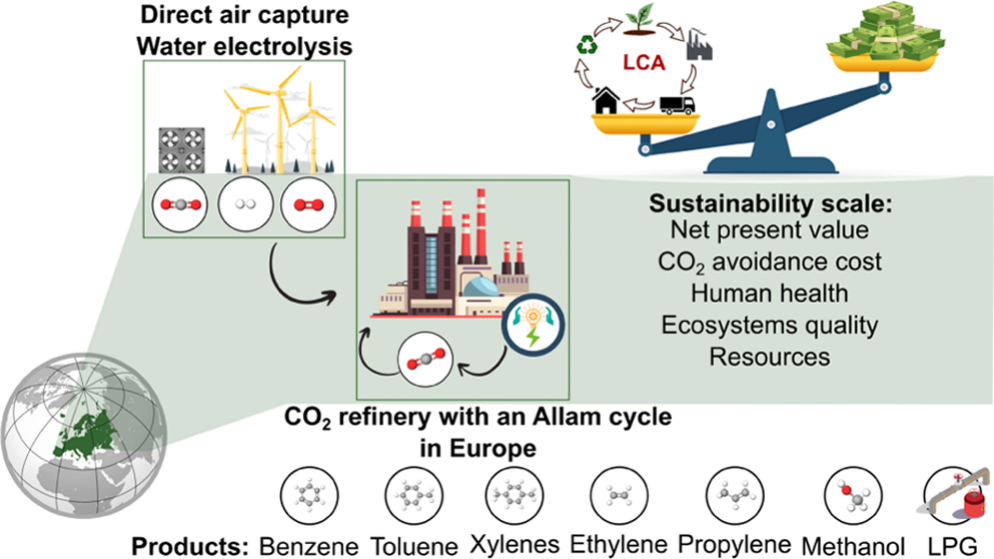

The consequences of global warming call for a shift to circular manufacturing practices. In this context, carbon capture and utilization (CCU) has become a promising alternative toward a low-emitting chemical sector. This study addresses for the first time the design of an integrated CO_2_ refinery and compares it against the business-as-usual (BAU) counterpart. The refinery, which utilizes atmospheric CO_2_, comprises three synthesis steps and coproduces liquefied petroleum gas, olefins, aromatics, and methanol using technologies that were so far studied decoupled from each other, hence omitting their potential synergies. Our integrated assessment also considers two residual gas utilization (RGU) designs to enhance the refinery’s efficiency. Our analysis shows that a centralized cluster with an Allam cycle for RGU can drastically reduce the global warming impact relative to the BAU (by ≈135%) while simultaneously improving impacts on human health, ecosystems, and resources, thereby avoiding burden-shifting toward human health previously observed in some CCU routes. These benefits emerge from (i) recycling CO_2_ from the cycle, amounting to 11.2% of the total feedstock, thus requiring less capture capacity, and (ii) reducing the electricity use while increasing heating as a trade-off. The performance of the integrated refinery depends on the national grid, while its high cost relative to the BAU is due to the use of expensive electrolytic H_2_ and atmospheric CO_2_ feedstock. Overall, our work highlights the importance of integrating CCU technologies within chemical clusters to improve their economic and environmental performance further.

## Abstract 2

Using life cycle assessment and techno-economic analysis, we compared different designs for a CO_2_ refinery with its fossil-based counterpart.

## Introduction

Meeting the Paris Agreement and limiting the global average temperature rise below 2 °C above pre-industrial levels^1^ will require tailored strategies for the different economic sectors along with collaborative actions. Besides, the production and use of energy across economic sectors contribute 75.0% of the EU’s total greenhouse gas (GHG) emissions,^2^ and thus, improving the energy source will indirectly enhance the performance of other sectors, i.e., the chemical sector consumes 10.0% of the worldwide energy demand.^3^ The chemical sector, regarded as a hard-to-abate sector yet to be decarbonized, could shift toward renewable carbon feedstock to curb its emissions. Notably, the production of chemicals is currently based on building blocks that are predominantly produced from fossil carbon in conventional crude oil refineries,^4^ namely, short-chain alkenes (ethylene and propylene) and monocyclic aromatics (benzene, toluene, xylenes [BTXs]). In 2020, the demand for ethylene and propylene amounted to 168 and 116 Mt, respectively, whereas the BTXs market was almost half of the olefins market (benzene 56, toluene 29, and xylene 46 Mt, respectively).^5^ Moreover, the production of short-chain alkenes will double between 2020 and 2040,^3^ due to the increasing need for goods and services, inevitably increasing the GHG emissions.

Alternatively, chemicals or fuels could be produced from carbon dioxide (CO_2_) following carbon capture and utilization routes (CCU). CCU could help reduce carbon emissions while creating economic value from CO_2_.^6−8^ Moreover, replacing fossil carbon feedstocks could circumvent impacts related to their extraction, transportation, storage, and use.^9^ Several CCU routes have been put forward based on thermo- and electro-catalytic processes, mainly focusing on C1-related products (e.g., carbon monoxide, methane, methanol, and formate),^10−12^ and, to a lesser extent, on C2–C3 chemicals (e.g., ethylene, ethanol, and propanol).^13,14^ Alternative substitutes or blending agents for fuels (e.g., dimethyl ether [DME]^15^ and oxymethylene dimethyl ethers [OME]^16^) also attract significant attention for CCU applications. However, the activation of CO_2_ requires a high amount of energy either directly or indirectly, e.g., the direct use of energy or a co-reactant with a high energy content (e.g., electrolytic hydrogen [eH_2_]^17^ via hydrogenation, or methane via dry reforming^15^), and specific infrastructure. Moreover, the CO_2_ source dictates the necessary amount of heat and power in the capture process, which is often large, and thus, its environmental footprint.^18^ In this regard, CCU based on fossil-based CO_2_ cannot help close the carbon loop since it will eventually be released after the chemicals’ life cycle, becoming a temporal storage solution.^6^ Alternatively, there are other end-of-life strategies for treating the CO_2_ supplied by the latter sources, e.g., mineralization and CCS.^19^ Moreover, CO_2_ could be captured from the air via direct air capture (DAC) units, although its currently high energy requirements are still a major obstacle.^20,21^

The previously mentioned bulk chemicals could be obtained via indirect CCU routes based on methanol (MeOH) synthesized from H_2_ and CO_2_, the specific origin of which will dictate the overall potential benefits. Besides, short-chain alkenes could be produced via the methanol-to-olefins (MTO) reaction, first introduced by Mobil Corporation in 1977,^22^ and commercialized in 2010.^23^ Currently, MTO production employs fossil carbon as the primary feedstock, like in China’s coal- and methanol-to-olefins (CTO and MTO, respectively) plants that seek to reduce oil dependency and exploit domestic coal resources. Furthermore, monocyclic aromatics could also be produced from MeOH via the already mature methanol-to-aromatics (MTA) process.^24^ Methanol as an intermediate could also enable producing products to substitute or blend fossil fuels, via (i) two stages of dehydration to gasoline (methanol-to-gasoline, MTG),^25^ (ii) dehydration to DME,^26^ and (iii) several pathways to OMEs.^16^ These schemes lie within the methanol economy concept introduced by Nobel Prize winner Olah.^27^ The latter transformations of methanol can arguably be perceived as the most important and mature link between the C1-CCU (e.g., MeOH) and petrochemicals or fuels until other pathways reach a similar maturity level, e.g., direct and selective conversion of CO_2_.^28−30^ We stress that for these routes to be environmentally friendly, they should avoid fossil-based feedstock.^31^

Given the critical role of the CO_2_ and H_2_ sources in CCU, they should be carefully optimized to reduce costs and impacts. This could be done by circular carbon pathways, which consider a closed use of carbon in various forms in several value chains, i.e., biomass and recycled plastics, among others,^6^ while also integrating direct utilization of captured CO_2_, and thus, avoiding the energy-intensive desorption step.^32^ For example, Meys et al.^33^ demonstrated that the circular carbon pathway, via utilizing biomass and CO_2_ from various sources combined with large-scale chemical and mechanical recycling, could simultaneously reduce the energy demand and operational costs relative to a fossil-based industry with carbon capture and storage (CCS). Namely, the authors considered CO_2_ captured from (i) chemicals and plastics production, (ii) waste incinerators, and (iii) DAC facilities, and excluded capture from fossil power plants. Furthermore, Jens et al.^34^ considered the capture of CO_2_ with methanol from raw natural gas of two different compositions and the subsequent utilization with H_2_ for their conversion to methyl formate. The authors concluded that the cost and environmental burdens could be reduced by using directly the mixture of absorbed CO_2_ and methanol, instead of following a two-step approach, i.e., CO_2_ adsorption–desorption and then CCU. Nonetheless, the design mentioned above could lead to benefits only when the solvent–product separation is less energy-intensive than the CO_2_ desorption step.

CCU routes are often assessed decoupled from each other, or other processes and industries, thus omitting attractive synergies that may increase their environmental and economic appeal. For example, the co-location with biomass processing plants could provide great benefits, including but not limited to the utilization of biogenic carbon (as captured CO_2_) and H_2_ from gasification.^33^ Another example could be the utilization of captured CO_2_ from integrated facilities instead of its storage, as an alternative to the integrated ethylene production plant based on shale gas and bioethanol dehydration considered by He et al.^35^ Furthermore, the integration of facilities usually offers great advantages, such as production logistics, integration opportunities, centralized energy supply, wastewater treatment, and waste disposal systems.

Notably, several CO_2_-based routes generate residual gas streams with high calorific value,^36,37^ so an effective residual gas utilization (RGU) strategy may lead to substantial savings, particularly using an Allam power cycle.^38^ These residual waste streams are the byproduct of utilizing captured CO_2_ and eH_2_. Therefore, this high-pressure power generation cycle, based on an oxy-combustion, coproduces pure CO_2_, which in turn could reduce, partly, the feedstock demand from alternative sources. At the same time, an Allam cycle operating in tandem with CCU, and water electrolysis could utilize partly or entirely the oxygen produced from the latter activity. An Allam cycle was successfully demonstrated in 2016 at a 50 MW_th_ test facility at La Porte, Texas, while a utility-scale project (≈300 MWe) is expected to be operational by 2026.^38,39^ In a recent study, Fernández-Torres et al.^40^ investigated the integration of oxy-combustion with CCU for high-quality gasoline production from atmospheric CO_2_ and H_2_, and compared it to the conventional alternative with a gas turbine along with additional heat recovery by the steam generator. They found that oxy-combustion cycles outperform conventional utilization in terms of mass and carbon efficiency.

Here, we design for the first time a rigorous CO_2_ refinery for methanol, olefins, aromatics, and liquefied petroleum gas (LPG) production. The refinery, which utilizes atmospheric CO_2_, encompasses three main synthesis and recovery steps: (i) methanol from CO_2_ hydrogenation (MeOH); (ii) olefins via methanol intermediate, i.e., MTO; and (iii) aromatics via methanol intermediate, i.e., MTA. Finally, we also evaluate two RGU designs, i.e., the Allam cycle and conventional heat and power recovery, and quantify the advantages of integrating CCU routes using life cycle assessment (LCA) and techno-economic analysis.

The remaining article is organized as follows. In Problem Statement and Scenarios Definition section, we briefly introduce the assessment scenarios of our study. Furthermore, Process Description and Economic Assessment section briefly describes (i) the process, (ii) the two residual gas utilization models, and (iii) the economic analysis. Life Cycle Assessment section provides the details of the LCA. Results and Discussion section discusses the LCA results for the CO_2_ refinery alternatives in Germany while also including a sensitivity analysis on the CO_2_ refinery location. In Results and Discussion section, we also provide the financial analysis results. Finally, we close with the Conclusions section.

## Problem Statement and Scenarios Definition

**Figure 1:**
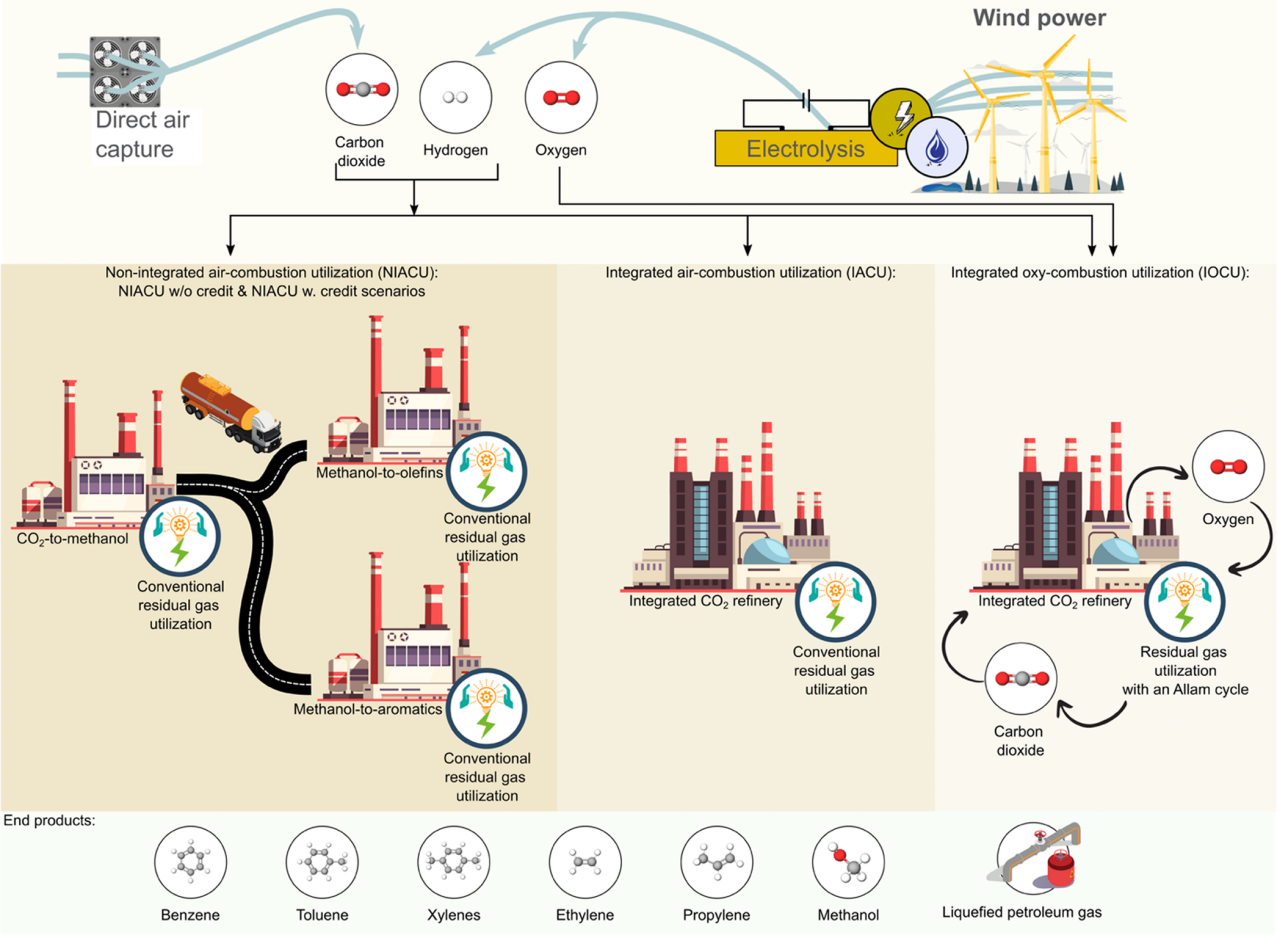
Schematic representation of the scenarios considered in this study.

Here we design a CO_2_ refinery and quantify the benefits of integrating the CCU methanol synthesis with MTO and MTA clusters, located in Europe, to exploit potential synergies of mass and energy integration, along with the advanced utilization of the residual gases. To carry out our analysis, we define four representative scenarios summarized in Figure 1.

In scenario 1, labeled as non-integrated air-combustion utilization (NIACU) w/o credits, energy integration is restricted because of the distance between facilities, so any excess energy from the RGU is wasted. The second scenario is similar to the first one; however, credits are granted for the excess energy assuming its use in a district or other industrial applications, e.g., NIACU w. credits. In the NIACU scenarios, methanol produced from CO_2_ and eH_2_ is transported to the MTO and MTA facilities. At the same time, each process is equipped with a conventional heat and power recovery cycle, e.g., air combustion. We consider methanol transport via lorry since, over the recent decades, the share of road transport has increased at the expense of rail for intra-EU activities.^41^ In particular, we assume a single methanol plant located 216 km from the MTO and MTA facilities The latter distance is equivalent to the average length with which a lorry distributes 79.3% of the total chemical mass in Europe,^42^ while train and barge are responsible for the remaining 14.7 and 6.0%, respectively. Furthermore, the intra-EU chemicals transport via lorry, for 216 km, may not be influenced by current EU policies aiming to shift 30% of road freight over 300 km to other means (i.e., rail or barge) by 2030, and more than 50% by 2050.^43^ In these scenarios, the methanol facility is in the middle of a hexagon, while the MTO and MTA units are in the edges, following a similar concept as in Liptow et al.^44^ Moreover, the methanol precursors, CO_2_ and H_2_, are supplied through DAC (powered by the national grid, while consuming natural gas heating) and wind-powered electrolysis using a polymer electrolyte membrane (PEM) electrolyzer. We assume that the location of the DAC and PEM units is the same as that of the methanol synthesis facility. Moreover, only one facility can be placed at each edge and an equal methanol consumption in the MTO and MTA process. Notably, sharing the methanol intermediate between the MTO and MTA processes equally is a modeling choice that simplifies the LCA implementation. The scope could be expanded to investigate the optimal share of MTO and MTA which can lead to lower total investment, within a similar framework as in Baliban et al.,^45^ which we will leave as future work.

In the third scenario, we consider a centralized cluster, where the residual streams are mixed and combusted with air to recover heat and power, with an acronym integrated air-combustion utilization (IACU). Finally, the fourth scenario considers a centralized CO_2_ refinery equipped with an Allam cycle, labeled as integrated oxy-combustion utilization (IOCU). Furthermore, the oxygen consumed in IOCU originates from the water electrolysis activity. The cluster with an Allam cycle generates power and a pure CO_2_ stream while requiring heating from natural gas as a trade-off.

## Process Description and Economic Assessment

This section discusses briefly the utilization of CO_2_ with eH_2_ toward olefins, aromatics, and LPG, which are eventually integrated into a single CO_2_ refinery. The methodology used in the economic assessment is also discussed. In the Supporting Information (SI) we provide the specific details of the developed simulation models, along with the detailed process flow diagram (see Figure S1 in the SI), and the cost parameters used in our analysis.

### CO_2_ Refinery Descriptions

We developed Aspen HYSYS process flowsheet(s) where the green MeOH process provides the feedstock for LPG, olefins, and aromatics production via CCU (Figure S1 in the SI). Methanol is synthesized with a commercial Cu–ZnO–Al_2_O_3_ catalyst and the reactor operates at 237–280 °C and 50.0 bar,^46^ from atmospheric CO_2_ and eH_2_. Subsequently, methanol is purified to 99.9% using two flash separators and one distillation column based on the work by González-Garay et al.^17^ Overall, 1.00 kg of methanol requires 1.43 kg of CO_2_ and 1.95 × 10^–1^ kg of H_2_ while generating 0.56 kg of wastewater as a nonvaluable byproduct. Furthermore, three residual gas streams (RGU 1–3, Figure S1 in the SI) are sent to the utilization cycle for the generation of energy, (and pure CO_2_ stream in the design with an Allam cycle), as discussed in the RGU subsection that follows. These streams consist of a purge from the first flash unit (44.3 bar), the second flash unit vapor outlet (1.8 bar), and the partial condenser’s vapor stream (1.0 bar), whose pressures are equalized before entering the burner of the RGU stage.

A portion of the generated MeOH is subsequently dehydrated at 1.5 bar and 450 °C over a zeolite catalyst, namely, SAPO-34, based on some previous work developed in Aspen HYSYS (Figure S1 in the SI).^37^ We utilize the released heat from the latter exothermic reaction in a two-stage Rankine cycle. The purification is based on cryogenic separation, and thus, the water is removed first to avoid the formation of hydrates. Subsequently, since the dry stream contains a small portion of H_2_, a series of three cryogenic knockout drums and a pressure swing adsorption unit are used for its recovery and purification. Finally, the valuable products are recovered via a sequence of distillation columns with 99.9 wt %. purity. Overall, the production of 1.00 kg of valuable aggregate products from the MTO step requires 2.40 kg of methanol, while 1.35 kg of wastewater is cogenerated as a nonvaluable byproduct, while three residual gas streams (remaining mass) are mixed to generate the RGU 4 stream (29.2 bar) that, as before, is sent to the utilization cycle for energy generation.

Finally, another Aspen HYSYS process flowsheet was developed, where the aromatics generation from methanol takes place at 4.0 bar and 475 °C (Figure S1 in the SI).^47^ As before, we utilize the reaction’s released heat in a two-stage Rankine cycle, and a high amount of coproduced water needs first to be removed, while the final products are purified using a series of distillation columns. Overall, the production of 1.00 kg of valuable aggregate products from the MTA step requires 2.34 kg of methanol, while 1.21 kg of wastewater is generated as a nonvaluable byproduct. Finally, a vapor stream primarily consisting of C2 and lightweight components (RGU 5, 17.0 bar) is sent to the RGU cycle, which is discussed next.

### Residual Gas Utilization

**Figure 2:**
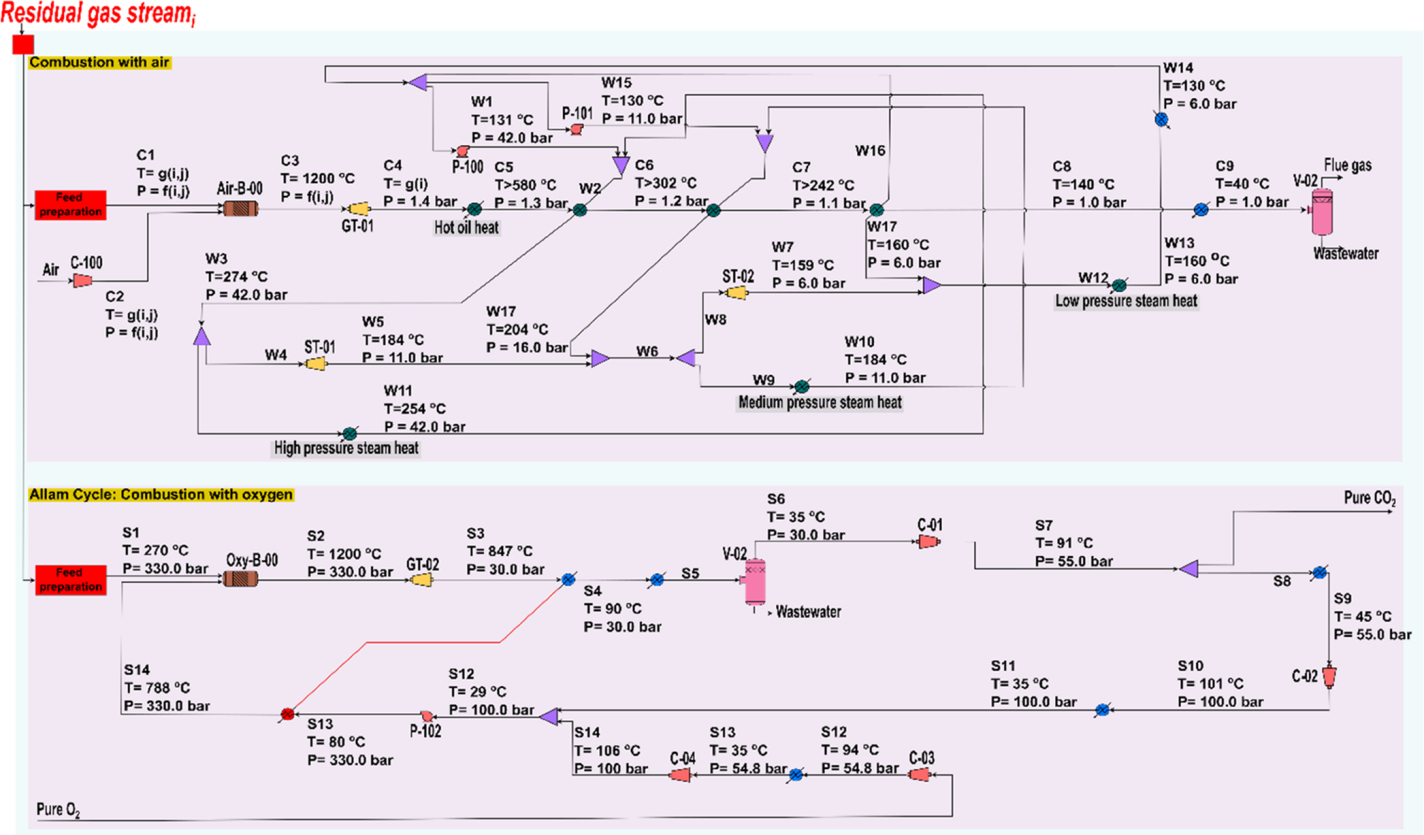
Process flowsheet of the conventional heat and power generation (top) and the Allam cycle (bottom) design.

We consider two RGU alternatives (Figure 2), also simulated in Aspen HYSYS, conventional heat and power recovery cycle with an air burner and an Allam cycle for power and CO_2_ generation. The combustion of the residual gases mentioned above, generated from CO_2_ and green eH_2_, provides a defossilized energy source. The best utilization route of the latter energy source, i.e., heat vs {electricity and CO_2_}, is dictated by the regional markets in which the plant operates.

Notably, the RGU streams are adjusted to the conditions of the burner, mixed, and subsequently fed into the utilization cycle. The RGU cycles are designed based on the conditions of the residual gas stream(s) of the respective facility and scenario (i.e., *T* = *g*(*i*,*j*) and *P* = *f*(*i*,*j*), where *i* represents the respective facility *i* for scenario *j*, Figure 2 (top) and Figure S1 in the SI) and the optimal heat exchange network (HEN) characteristics (Figure S14 in the SI). Notably, the burner for the RGU for the MeOH step, and for both NIACU scenarios, operates at 1.0 bar, and 29.2 and 17.0 bar for the MTO and MTA processes, respectively. At the same time, in IACU, the air burner operates at 17.0 bar, while in IOCU, the oxy-burner operates at 330 bar. The selection of these operating conditions is based on the energy requirements for equalizing the pressure among the available RGU streams.

In the conventional heat and power recovery cycle (Figure 2, top), the air feedstock is compressed and subsequently co-fed with the RGU into the burner (Air-B-00). The flow of air is adjusted accordingly to keep the temperature of the flue gas stream at 1200 °C to avoid extreme operating conditions. The flue gas pressure is then released to 1.4 bar in a gas turbine (GT-01), and subsequently, the outlet stream is used to generate electricity, low-, medium-, high-pressure steam (LPS, MPS, and HPS, respectively) and hot oil. The generation of utilities in the RGU cycle is based on the HEN design. Finally, if the utilization cycle cannot deliver the HEN’s targets, additional natural gas heating is considered.

In the Allam cycle of the CO_2_ refinery (Figure 2, bottom), along with the residual gases, a stream of CO_2_ and O_2_ is co-fed into the burner (Oxy-B-00), which operates at 330 bar. The CO_2_ in the feed acts as inert gas for the oxy-combustion burner to keep the flue gas temperature at 1200 °C, as done by N_2_ in the air burner. Subsequently, the flue gas pressure is released to 30 bar in a gas turbine (GT-02). The heat of the flue gas stream is used to preheat the O_2_ and CO_2_ inlet mixture of the oxy-burner. The flue gas is then cooled to 35 °C, and water is removed via a flash separation unit (V-02), which leads to a pure CO_2_ stream (99.9 wt %) at the gaseous outlet. Subsequently, the vapor outlet is recompressed to 55 bar and partly recycled to the methanol synthesis facility, while the remaining is compressed further to 100 bar and liquefied via cooling to 35 °C. Notably, the oxy-burner can utilize the electrolysis oxygen co-product, eO_2_, which enters at 30 bar into the cycle and is compressed to 100 bar in two stages. Finally, the latter liquid O_2_ and CO_2_ streams are mixed, and their pressure is increased to 330 bar with a pump.

### Financial Analysis

The methodology used to estimate the CO_2_ refinery’s costs follows the standard procedure for preliminary estimates. Namely, the methodological steps, correlations, and factors used are available in Towler and Sinnott (Chapters 7–9).^48^ The process simulator provides the equipment units’ sizes, the material and energy flows needed in estimating the revenues, capital expenditures, and finally, the variable and fixed operational costs (FOC). The key economic indicator of this study is the net present value (NPV), which is calculated based on the following parameters: (i) 30 year plant lifetime, (ii) 8000 h year^–1^ of operation, (iii) a 7% interest rate, (iv) a 30% federal income tax, and finally, (v) 7 year MACRS depreciation charges. Moreover, in our analysis, we used global averaged costs for materials and utilities and prices for the valuable products while conducting a sensitivity analysis to tackle their variability. Finally, we provide in the SI the cost parameters used in this study.

## Life Cycle Assessment

LCA is a holistic methodology to analyze the environmental impact of products, processes, and services and rank alternatives relative to representative benchmarks.^49^ We here quantify the environmental performance following the ISO standards (ISO:14040 and 14044),^50,51^ as described next.

### Goal and Scope Definition

Our analysis delves into the performance of CO_2_ refining, where we pursue three goals based on the four representative scenarios described in Problem Statement and Scenarios Definition section (Figure 1). In goal 1, we aim to evaluate the performance of CO_2_-based products relative to their fossil-based counterparts. Goal 2 aims to quantify the potential benefits of decentralized and centralized CO_2_ refining clusters, along with the comparison between two RGU designs. Moreover, in goal 3, we aim to identify the regional influence of the investigated centralized RGU designs, which in short requires the consideration of the domestic character for the heat and electricity markets, wind turbines load hours, and the respective needs of the underlined designs. Goals 1 and 2 are assessed by having Germany as the geographical scope (base case), whereas several locations within Europe are studied for the sensitivity analysis of goal 3.

We, here, consider as a functional unit (FU) the joint production of valuable compounds of the designed CO_2_ refinery. Namely, the FU is the production of ethylene (1.00 kg), LPG (0.90 kg), propylene (0.66 kg), mix-xylenes (0.45 kg), pentane (0.24 kg), methanol (0.22 kg), butene (0.20 kg), and benzene (0.06 kg). The latter valuable product distribution is based on the yield of the various synthesis steps of the CO_2_ refinery. Hence, an optimal utilization of feedstock in the MTO and MTA steps might lead to better performance. However, this modeling choice simplifies the implementation of the LCA. The joint FU was selected to avoid allocation decisions since alternative approaches will lead to different burdens depending on the weight factors. For example: (i) in economic allocation, the price of products, and their correlation, might change, or some products might not have a market (C_9+_ aromatics), (ii) in energy allocation, only LPG and methanol can be considered as energy carriers, while (iii) in mass allocation, all products will be considered based on their direct output. The joint FU nevertheless avoids this step, while describing the same underlying trend. At the same time, any allocation methods can be applied by multiplying with the appropriate allocation factor. Finally, we omitted the cogenerated C_9+_ aromatics in the FU of our assessment (i.e., they are assumed to carry no burden) since their further transformation, via toluene or benzene, is needed for delivering valuable products, e.g., xylenes.^52^

All in all, the system boundaries cover the water splitting, MeOH, MTO, MTA, and DAC processes. We adopt a cradle-to-gate scope and an attributional approach. Thus, we included all of the upstream activities while omitting the products’ downstream transformation, assuming identical downstream processes. Finally, for both NIACU scenarios, the methanol feedstock is supplied via inland transport based on the assumptions available in the literature, considering an average transportation distance equal to 216 km as discussed previously.^42^

### Life Cycle Inventory (LCI)

The LCIs of the conventional products, which are used as the benchmark, were obtained from ecoinvent v3.5.^53^ Thus, we consider their production from either cracking processes (olefins and aromatics), petroleum refining (LPG), and natural gas steam methane reforming (methanol). Furthermore, the CO_2_ refinery’s mass and energy flows were retrieved from the Aspen HYSYS simulations, discussed in the previous section (foreground system), with data from ecoinvent v3.5 (background system).^53^ In addition, we consider the generation of cryogenic and hot utilities to satisfy the internal needs of the facilities.^54^ At the same time, we assumed cooling water utility as a 50–50% once-through and a recirculating cooling system, describing the evaporation loss as a percentage of the cooling water requirements. We, here, use as a reference process for the cooling water evaporation losses of the methanol production the corresponding activity in the ecoinvent database (see SI).^53^ The LCIs for the electrolytic H_2_ feedstock^55^ and CO_2_ feedstock^20^ are based on literature sources and the ecoinvent database.^53^ Notably, we assume that DAC supplies the CO_2_ feedstock of the refinery, whereas the eH_2_ is provided by wind-powered electrolysis. Furthermore, the national grid satisfies the inputs for all activities of the refinery, except for the PEM electrolyzer. We provide the latter LCIs in the SI.

### Life Cycle Impact Assessment

We quantify the impact of the CO_2_ refinery with the three endpoints and eighteen midpoint LCA indicators of the ReCiPe 2016 (H) methodology.^56^ Namely, we consider endpoint impacts on human health (HH) expressed in disability-adjusted life years (DALYs), ecosystem quality (EQ) quantified in local species loss integrated over time (species *y*), and resource depletion (RD) in USD ($). The latter indicates the premium involved in future mineral and fossil resource extraction due to the current exploitation of resources. Notably, the endpoint categories are linked to the eighteen midpoint indicators of ReCiPe 2016 using a weight factor per impact category. Finally, we provide in the manuscript the impact of global warming (GW), which quantifies the kgCO_2eq_ emitted, while we provide in the SI the results for the remaining midpoints indicators of ReCiPe 2016.

## Results and Discussion

We first discuss the GW impact of the CO_2_ refinery’s function operating in Germany, then describe the overall burdens on HH, EQ, and RD for the same location. Subsequently, we evaluate the CO_2_ refining performance in HH, EQ, and RD for the two centralized RGU pathways in different geographical regions. Finally, we discuss key financial indicators for the designed production clusters.

### Environmental Assessment

#### Global Warming Impact

**Figure 3:**
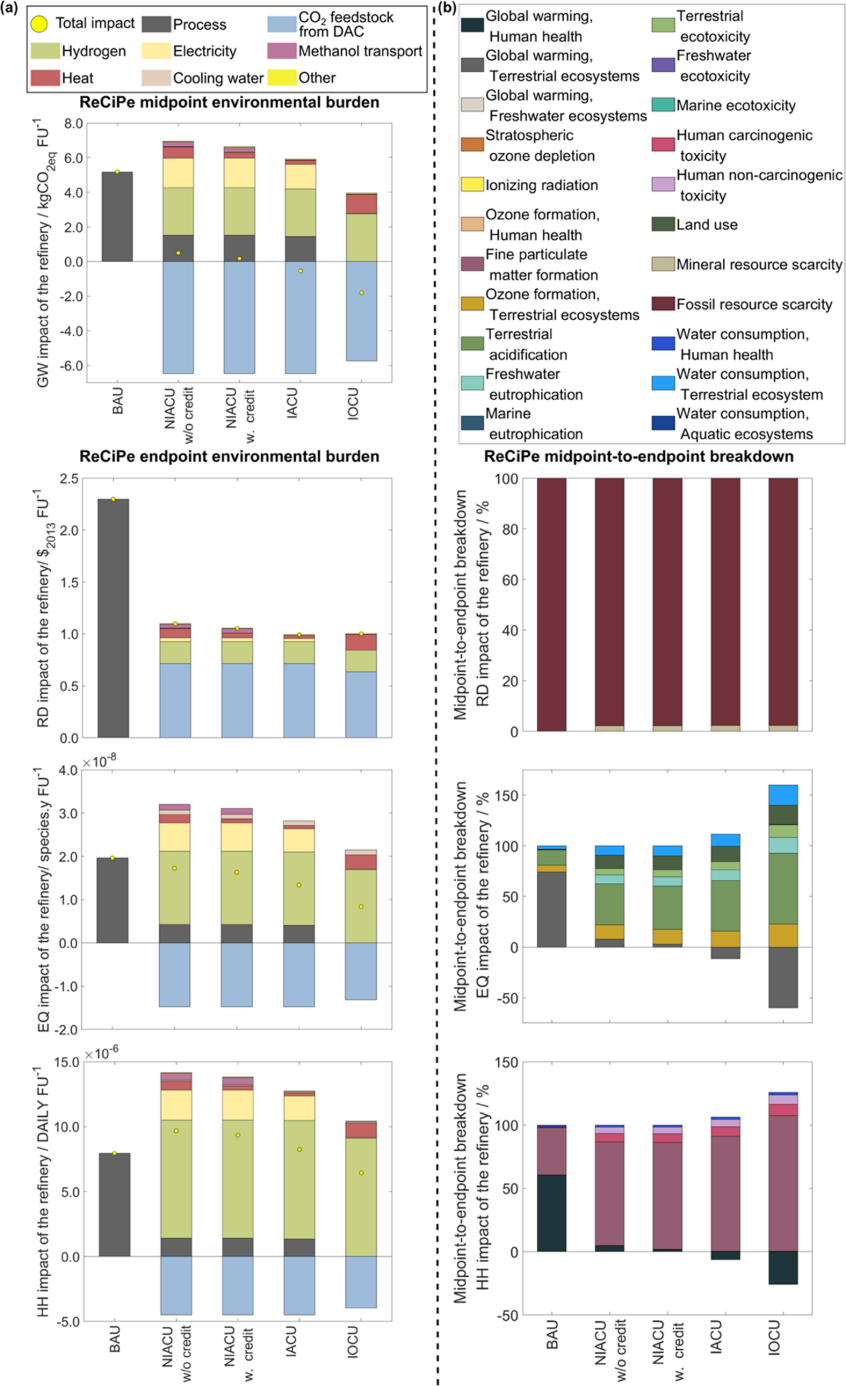
(a) Environmental impact on the ReCiPe midpoint of global warming (GW), and the three ReCiPe endpoints (i) resources depletion (RD), (ii) ecosystems quality (EQ), and (iii) human health (HH) of the CO_2_ refinery per FU for the scenarios described in Figure 1. The net impact is depicted as a yellow dot, which is the sum of all contributors. The positive stacks correspond to burdens, whereas negative stacks represent benefits emerging from the utilization of atmospheric CO_2_. (b) Midpoint-to-endpoint breakdown toward RD, EQ, and HH. Finally, in Figure S7 in the SI, we provide a detailed activities breakdown for each midpoint category.

In terms of GW, we find that in all of the scenarios the production facilities located in Germany outperform significantly the business-as-usual (BAU), which exhibits an impact of 5.2 kgCO_2eq_ FU^–1^ (Figure 3, top left). Notably, the burden lies within the 90.5, 96.7, 110.6, and 134.6% range lower than the BAU for NIACU w/o and w. credits, IACU, and IOCU, respectively. The latter competitiveness originates from the use of atmospheric CO_2_ (via DAC) to a great extent, while avoiding at the same time fossil-based emissions (e.g., from feedstock). In addition, the refining of CO_2_ performs best when switching from decentralized to centralized designs, with IOCU (RGU with an Allam cycle) having the lowest footprint. Furthermore, considering environmental credits in the decentralized design only leads to a slight GW improvement. The latter outcomes emerge due to (i) the transportation of methanol in the decentralized scenarios (NIACU), (ii) the more efficient use of energy in NIACU w. credits and IACU, and (iii) the more efficient power generation in IOCU compared to IACU. Besides, IOCU’s electricity requirements are significantly reduced while increasing the need for heating. At the same time, the heating is partly satisfied from the RGU in the decentralized MeOH and MTO plants (16.0 and 77.0%, respectively), while the MTA facility has a 1.9-fold excess. Notably, centralized manufacturing with conventional RGU could satisfy the cluster’s heating by 79.2%, while IOCU can fulfill 98.4% of the electricity (excluding indirect needs, i.e., eH_2_ and CO_2_) via the Allam cycle. Finally, by removing the burdens of the methanol transport activity in NIACU w/o and w. credits, we observe that the GW improvements relative to the BAU become 95.5 and 101.7%, respectively, and thus, are still lower than in the integrated designs.

The LCA breakdown reveals that the main impact contributor is eH_2_ (2.7 kgCO_2eq_ FU^–1^), where there is marginal potential for improvements since we assume a high energy conversion efficiency (80.0% of the lower heating value). The high contribution of eH_2_ to the life cycle emissions emerges due to the significant consumption of power (41.7 kWh kg^–1^ for the assumed efficiency) and embodied burdens associated with the windmills and electrolyzers supply chains. Direct electricity supply from the national grid follows ({1.4–1.7}, and 3.4 × 10^–2^ kgCO_2eq_ FU^–1^, for {NIACU w/o and w. credits, and IACU} and IOCU, respectively), while the burden from heating plays a less significant role in the conventional RGU scenarios (0.6, 0.3, 0.2, and 1.1 kgCO_2eq_ FU^–1^, following the same sequence). The methanol transport activity in NIACU scenarios leads to a GW impact of 2.6 × 10^–1^ kgCO_2eq_ FU^–1^, significantly smaller than the other contributors mentioned above. The utilization of atmospheric CO_2_ in the refinery significantly benefits the GW impact (−6.5 and −5.7 kgCO_2eq_ FU^–1^). At the same time, the total GW impact savings due to the use of atmospheric CO_2_ are higher than the total burden of the conventional counterpart in all scenarios. Even though DAC seems a practical way forward for the chemical sector, we note that according to Müller et al.,^18^ at present, supplying CO_2_ from other sources (e.g., ammonia or ethylene oxide plants) could lead to even higher benefits in the GW impact than using DAC. Notably, the CO_2_ feedstock that leads to the latter benefit is lower in IOCU (13.4 and 11.9 kgCO_2_ FU^–1^ for {NIACU w/o and w. credits, and IACU} and IOCU, respectively). Besides, the recycled amount is equivalent to 11.2% of the total feedstock while eliminating direct process emissions. Hence, since the capture of atmospheric CO_2_ has a relatively high carbon intensity (−0.5 kgCO_2eq_ kgCO_2_) due to the German grid, there is a higher incentive to avoid the chemical cluster’s direct emissions and reduce the energy requirements via RGU with an Allam cycle.

#### Overall Environmental Performance

We observe that CO_2_ refining improves the RD and EQ impact for all of the scenarios (Figure 3, left); however, there is burden-shifting toward HH in most cases. The burden reduction is more drastic in the former (52.1–56.7%) and smaller and in a broader range in the latter (12.0–17.0 and 31.7–57.2%, low- and high-end in both NIACU and IACU–IOCU scenarios, respectively). Conversely, NIACU w/o and w. credits led to a worsening of HH by 21.8–17.6%. Furthermore, even though the IACU led to further burden reductions in EQ and RD, it failed to outperform the fossil-based products in HH (3.8% higher than the BAU). Finally, the transition to IOCU leads to a win–win–win (HH–EQ–RD) scenario relative to the BAU since it attains an 18.9% reduction in HH while achieving the lowest EQ burden and RD similar to the other designs. Note that the latter behavior depends on the national grid’s performance, and thus, we address this effect later in a sensitivity analysis.

The RD breakdown of activities highlights the significance of the CO_2_ feedstock in the total impact (∼64%), followed by eH_2_, electricity, and heating. Furthermore, we observe that the HH and EQ benefits emerge using atmospheric CO_2_ that counteract the high burdens from other activities, such as the eH_2_, energy inputs, and direct emissions. Notably, we expect that, in the future, these benefits will be more pronounced due to improvements in the energy sources and DAC units.^20^ Finally, we observe an additional burden in the EQ metric due to cooling water in the manufacturing facilities, while this contribution is insignificant in HH and RD.

The midpoint-to-endpoint breakdown (Figure 3, right) shows that, as expected, the former, according to their respective weight factor, drive the occurrence of burden-shifting in the latter. Focusing on HH, the CO_2_ refinery’s GW contribution to this endpoint, unlike the BAU, is negligible and even provides a positive effect for the integrated designs. However, particulate matter formation and toxicity share increase drastically due to the vast energy consumption to generate eH_2_ and capture CO_2_. At the same time, for EQ, the worsening of terrestrial ecotoxicity, ozone formation, terrestrial acidification, land use, and water consumption categories, among others, counteracts the benefits obtained in the GW indicator due to the utilization of atmospheric CO_2_. The latter burden-shifting, as before, also occurs due to extensive energy consumption. Finally, although mineral resource scarcity rises significantly for the CO_2_ refinery, the scarcity of fossil resources remains the primary contributor to the RD endpoint. Besides, the PEM electrolyzer’s wind power increases significantly in the former midpoint, while the energy use for DAC contributes considerably to the latter. We provide the detailed activities breakdown for the eighteen midpoint categories of the ReCiPe 2016 method in Figure S7 in the SI.

#### Sensitivity Analysis on the Plant’s Location

**Figure 4:**
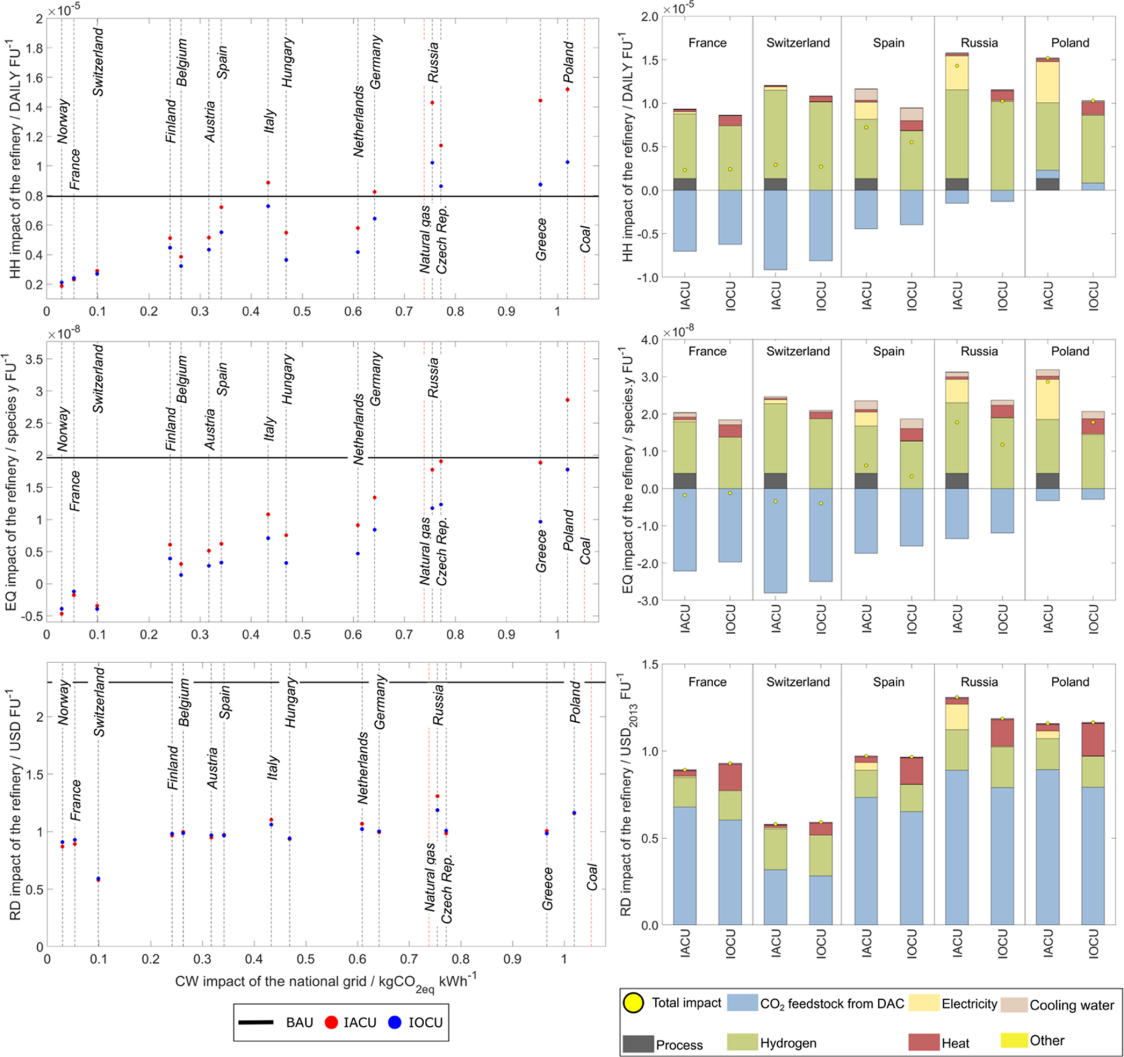
Environmental burden on human health (HH), ecosystems quality (EQ), and resources depletion (RD) of the CO_2_ refinery per FU in different regions of the world vs the global warming (GW) impact of the local power grid (left); and their breakdown (right).

The plant’s location sensitivity analysis reveals that the two centralized RGU perform equally in HH (Figure 4) and outperform the BAU in countries with a “clean” power grid (<0.15 kgCO_2eq_ kWh^–1^, such as Norway, Switzerland, and France). The latter is not the case in locations with higher-emitting national grids (>0.15 kgCO_2eq_ kWh^–1^), where the IOCU design is always more appealing than IACU. For regions with “milder” carbon-intensive grids (0.2 ≤ *x* ≤ 0.6 kgCO_2eq_ kWh^–1^), only in a few locations are both designs significantly better than the BAU (e.g., Finland, Belgium, and Hungary). In contrast, placing the refinery in Italy or Spain can lead to higher stresses due to the higher water consumption impact for the same amount of cooling, along with lower CO_2_ feedstock benefits due to the local energy markets (Figure 4, breakdown). At the same time, neither design is better than the BAU for locations with a “dirty” electricity grid (>0.7 kgCO_2eq_ kWh^–1^). In the EQ metric, we observe similar behavior as in HH. However, the CO_2_ refining products are more appealing in all investigated regions for grids with carbon intensity lower than 1.0 kgCO_2eq_ kWh^–1^. Notably, the cooling water contribution is more prominent than in the base case (i.e., Germany). Moreover, when the cluster is placed in either Russia, the Czech Republic, or Greece (electricity grids with GW, 0.7 ≤ *x* ≤ 1.0 kgCO_2eq_ kWh^–1^), only IOCU outperforms significantly the BAU in the EQ metric. Furthermore, with the current national grid characteristics, only the IOCU design can lower EQ burdens in Poland. Finally, the RD performance varies slightly in the different locations and is consistently lower than the BAU. Since the total impact is significantly lower than the fossil-based counterpart, their comparison could be neglected.

We can derive conclusions from the discussed trends linked to domestic characteristics (Figure 4, breakdown). Namely, the impact of wind-powered eH_2_ due to the difference in the turbines’ full load hours (Spain vs Russia), along with the stresses on water availability (Spain vs Switzerland), the burdens associated with extracting and transporting natural gas for heating, among other inputs supplied in the local market (France vs Switzerland), the national grid performance (France vs Poland), and overall the local stresses could significantly affect the appeal of CO_2_-based products. Besides, the eH_2_ and CO_2_ feedstocks are highly influenced by electricity and heat markets due to their high consumption (e.g., DAC requires 250 and 1750 kWh kgCO_2_^–1^ of electricity and heat,^20^ respectively). The regional characteristics become insignificant in regions with clean power grids, whereas the Allam cycle’s benefits become much more prominent otherwise.

### Economic Performance

We now extend the study to the economic indicators. Namely, we start by analyzing the (i) total fixed capital investment (TFCI), (ii) variable operating costs (VOC), and (iii) fixed operational costs (FOC). Finally, we provide the financial analysis based on the NPV, highlighting the bottlenecks of CO_2_-based production.

#### Capital and Operational Expenses

Our economic analysis shows that, at the current state, CO_2_ refining is economically unappealing. Notably, the revenues cannot cover the annual expenses (Table 1). We further observe that economies of scale reduce the TFCI by 1.6% when shifting from the NIACU scenarios to its integrated counterpart, while also avoiding the additional capital expenses related to the methanol transportation units. Regarding IOCU, the TFCI is higher by 0.5–2.1% than NIACU and IACU, respectively. At the same time, the total operating costs differences are marginal (0.9, 2.9, and 3.5% lower compared to NIACU w/o credits for its equivalent with credits, IACU, and IOCU, respectively).

**Table 1:** Investment and Operational Requirements and Revenues for the Investigated CO_2_ Refinery, and Associated NPV

	NIACU	NIACU		
	w/o credits	w. credits	IACU	IOCU
Capital (M$)				
TFCI	723	723	712	727
WC	83	83	82	83
transportation units	5	5	0	0
Operational (M$ year^–1^)				
VOC	932	923	916	910
FOC	26	26	25	26
transportation	11	11	0	0
Revenues (M$ year^–1^)				
	491			
NPV (M$)				
	–6958	–6841	–6585	–6528

Notably, on average, 96.0% of the VOC are connected to raw materials, while the utilities contribute 4.0% (Figure S5 in the SI). The eH_2_ feedstock, having a significantly higher price than its fossil counterpart ($3000 vs $1250 t^–1^),^57^ contributes on average 82.1% of the total raw materials costs, while the remaining is due to CO_2_. Regarding utility costs, heating contributes 41.0, 25.6, 22.2, and 91.9% of the total for NIACU w/o and w. credits, IACU, and IOCU, respectively. At the same time, the summation of electricity and heating costs comprise ∼95% of the total utility costs. All in all, the operational differences between scenarios render insignificant compared to the economic burden of eH_2_.

The compressors of the CO_2_ refinery are responsible for more than 55.0% of the TFCI, followed by heat exchangers (∼17.0%) and the reactors and turbines, which contribute, on average, 6.7% each. Moreover, the separation columns are responsible for almost 5.7% of the TFCI. Finally, the capital expenses for turbines and compressors for the IOCU design are higher by 27.0 and 2.3%, respectively, compared to the IACU (see Figure S5 in the SI).

#### CO_2_ Avoidance Cost

The economic assessment discussed above disregards the climate benefits of the CO_2_ refinery. For that reason, we calculate the CO_2_ avoidance cost, which could be interpreted as the tax on GHG emissions at which the production cost of a design with CO_2_ mitigation is the same as the fossil reference,^58^ e.g., the CO_2_ refinery, which is as follows^59^
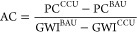
where AC is the CO_2_ avoidance cost (in $ tCO_2eq_^–1^), PC is the production cost (in $ FU^–1^), and GWI is the global warming impact (in tCO_2eq_ FU^–1^).

**Figure 5:**
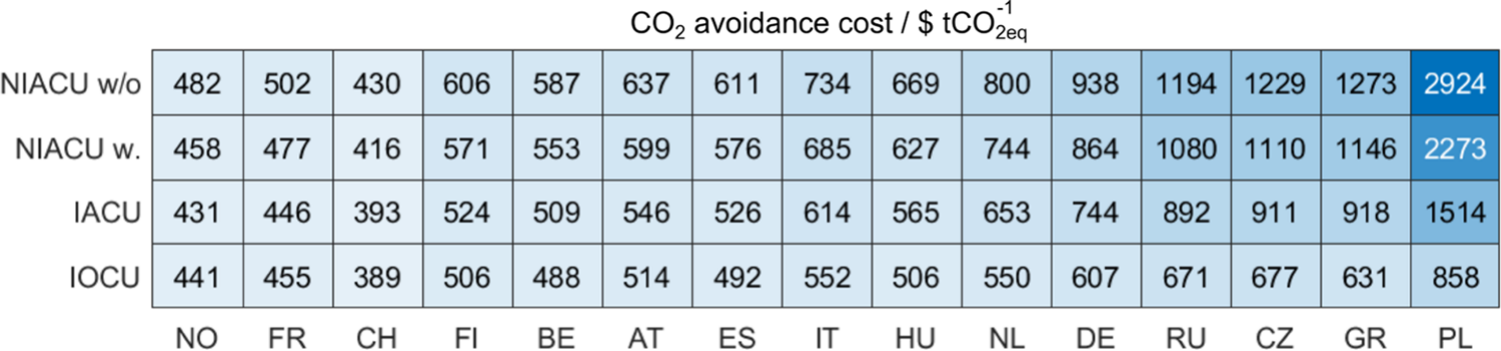
CO_2_ avoidance cost for the different locations considered in Figure 4. As a simplification, we use the same total annualized cost calculated for all countries based on average prices.

Figure 5 provides the AC calculated for the different locations considered in Figure 4, where the obtained range of values is consistent with intervals reported in the literature.^17,59^ Notably, the highest benefit per $ spent is obtained by placing the CO_2_ refinery in Switzerland, while Poland is the least favorable region. Nonetheless, the calculated ACs are significantly higher than 2022 carbon tax rates, which currently, range between $1 and $137 tCO_2eq_^–1^ in Poland and Uruguay, respectively, while Liechtenstein, Sweden, and Switzerland follow with a rate amounting to $130 tCO_2eq_^–1^.^60^

#### Financial Analysis

Table 1 provides the NPV for manufacturing the valuable products at the reference prices mentioned in Section 4. The results indicate that CO_2_-based manufacturing leads to negative NPVs. We further observe that IOCU obtains slightly less negative NPV than IACU, meaning that the RGU with the Allam cycle is more attractive. Since the operation of the CO_2_ refinery is unprofitable within the assumed financial factors and prices, we carry next a sensitivity analysis to identify the conditions under which the refining of CO_2_ becomes economically appealing.

#### Sensitivity Analysis on NPV

We consider a possible price decrease for eH_2_ and CO_2_ amounting to 50 and 100%, respectively. Besides, locations with attractive solar and wind availability and integrated energy systems could attain eH_2_ for under $2000 t^–1^ in the near future.^57^ Furthermore, subsidies and technological improvements for DAC could reduce the cost of CO_2_ substantially, while CO_2_ captured from point sources can be used at a lower cost. The supply of pure CO_2_ free of charge could reflect the (i) upgrade of biogas to bio-CH_4_ or (ii) H_2_ purification before the Haber–Bosch process without an integrated urea facility to utilize this CO_2_ stream. At the same time, CO_2_ taxes, and other geopolitical stressors, could trigger the price increase of goods, and thus, we assume a +50% variation. We further investigate the influence of the TFCI and the cost of electricity and heating (±30%). Finally, we omit varying the transportation distance of the methanol synthesis facility in NIACU w/o and w. credits, since the capital and operational costs, amounting to $5 million and $11 million year^–1^ (Table 1), respectively, represent a small percentage of the total expenses (0.6 and 1.1%, following the same sequence as before).

We find that the CO_2_ refinery’s NPV is significantly influenced by decreasing the price of eH_2_ and CO_2_ and increasing the revenues, i.e., products’ price (see Figure S6 in the SI). In the extreme cases, (i) eH_2_ with a price of $1500 t^–1^, (ii) free CO_2_, and (iii) a 50% increase in revenues, the NPV could become, in turn, {2.9–3.4}-fold, 1.4-fold, and {1.8–1.9}-fold lower compared to the base case, respectively. On the contrary, the expenses related to TFCI, and utilities (electricity and heating) affect only marginally the NPVs. Nonetheless, our results indicate a higher slope when varying the TFCI, while the trend for electricity and heating is scenario sensitive.

#### Breakeven Production

CO_2_ refining could become economically viable under favorable conditions based on the observations mentioned above. Thus, we provide in Table 2 two examples under which the CO_2_ refinery could attain economic profitability (NPV ≥ 0). In example one, we assume an eH_2_ and CO_2_ price of $1500 and $36 t^–1^, respectively, a 10% lower cost of electricity and a 15.6% increase in product prices. The latter eH_2_ cost represents the above-mentioned near-future optimistic estimate,^57^ while the CO_2_ feedstock cost is comparable to the capture cost in coal power plants and slightly higher with that captured from natural gas plants, which amounts to $36–53 and $48–111 tCO_2_^–1^, respectively.^61^ Notably, DAC technologies should undergo substantial improvements to attain the latter CO_2_ feedstock cost. Based on such improvements, IOCU breaks even, while IACU becomes profitable (NPV = $51 million). Moreover, using the GW impact for a CO_2_ refinery located in Germany, the CO_2_ avoidance cost ranges from $121 to $199 tCO_2eq_^–1^, and thus, for the integrated designs is close to the upper limit of the carbon tax rates mentioned previously.

**Table 2:** Key Parameter Changes for Achieving Economic Viability, i.e., NPV ≥ 0

NIACU w/o | NIACU w. IACU | IOCU	base case	breakeven example 1	breakeven example 2
NPV (M$)	–6958 | −6841	–249 | −153	–198 | −104
	–6585 | −6528	+ 51 | 0	+99 | 0
CO_2_ avoidance cost Germany ($ tCO_2eq_^–1^)	937 | 863	199 | 172	96 | 76
	743 | 606	139 | 121	55 | 57
CO_2_($ t^–1^)	90	36	0
eH_2_($ t^–1^)	3000	1500	1500
revenues (M$ year^–1^)	487	563	504
electricity ($ MWh^–1^)	70	63	63

In the second example, we assumed that the CO_2_ is supplied free of charge while keeping the eH_2_ and electricity prices the same as in example one. In such a case, to break even in IOCU, the revenues should increase only by 3.5% (where IACU has NPV = $99 million). Notably, the CO_2_ avoidance cost is even lower in this example, amounting to $55–96 tCO_2eq_^–1^. Hence, both examples highlight that the Allam cycle is unappealing when the CO_2_ price is low, which may be a limiting factor in the decision-making process. At the same time, the CO_2_ avoidance cost could become equivalent to the carbon taxation rates already imposed in Europe, and thus, the CO_2_ refinery may act as a promising transition pathway.

## Conclusions

This work performed a comparative life cycle and financial analysis of two decentralized and two centralized scenarios for CO_2_-based manufacturing of valuable products. Within our work, we investigated two appealing strategies for residual gas utilization (RGU) in carbon, capture, and utilization (CCU), i.e., (i) a conventional air burner to generate heat and power and (ii) a state-of-the-art Allam cycle to generate power and CO_2_. The refinery aims to coproduce olefins, aromatics, and liquefied petroleum gas based on the methanol economy and CCU concepts. Our environmental assessment covers (i) 18 midpoint categories, e.g., global warming (GW), and (ii) 3 overall performance metrics, e.g., impact on human health (HH), ecosystems quality (EQ), and resources depletion (RD). Finally, our study analyzed the key economic factors of CO_2_ refining.

Regarding the GW midpoint category, CO_2_ refining significantly reduces greenhouse gas emissions compared to the fossil BAU counterpart. However, the environmental analysis also highlighted a worsening of HH impacts while attaining substantial improvements in EQ and RD for three out of the four analyzed scenarios. Furthermore, the comparison between decentralized and centralized CO_2_ refining showed that the latter is environmentally superior. The centralized design with an Allam cycle led to a win–win–win scenario regarding the overall performance metrics. Such configuration acts as an opportunity for a circular concept in CCU applications by recycling pure CO_2_. It can also significantly improve the environmental performance of the manufacturing cluster in regions where the national grid has high carbon intensity, >0.15 kgCO_2eq_ kWh^–1^. Besides, a sensitivity analysis of the manufacturing location provided further insights into the performance of the two centralized RGU designs, indicating their suitability according to the environmental burdens of the regional energy system. The oxy-combustion cycle is more appealing for locations where the capture of atmospheric CO_2_ leads to higher burdens due to the performance of the regional energy grid. In contrast, the two RGU designs lead to similar overall environmental burdens under conditions of a clean national grid.

The financial analysis revealed that the investigated scenarios of the CO_2_ refinery are unprofitable, while the integrated design led to a marginal benefit by capitalizing on a common conventional RGU cycle, economies of scale, and avoiding the transport of intermediates. An integrated conventional RGU design is economically less appealing than an Allam cycle equivalent for the reference CO_2_ price ($90 t^–1^), while this behavior shifts when the latter cost decreases. Finally, while the profitability of CCU is highly linked to the precursors’ cost (H_2_ and CO_2_), under favorable conditions, small premiums in the prices of the products could accelerate the industrial implementation of a CO_2_ refinery.

This study identifies the main life cycle and financial implications for decentralized and centralized scenarios for a CO_2_ refinery, RGU strategies, and operation in several regions. Furthermore, our results highlight the significant role played by the RGU approach and the Allam cycle in the environmental performance of CO_2_-based products. Therefore, the RGU strategy in centralized CCU plants can aid the gradual decarbonization of chemical production without additional stresses of high-impact regional energy utilities. All in all, future technological improvements should aim to increase the economic appeal of an integrated CO_2_ refinery to complement its better environmental performance. At the same time, we suggest an optimization-based assessment and a product portfolio expansion to include fuels (gasoline, DME, and OMEs via methanol intermediate) to identify the best design of a CO_2_ refinery.

## Supporting Information Available

Modeling details for the processes modeled in Aspen HYSYS, life cycle inventories, and additional environmental and economic assessment results (PDF)
