# Supplementary material for: Economic and Environmental Performance of an Integrated CO2 Refinery
Source: ACS Sustain Chem Eng. 2023 Jan 26;11(5):1949–61. doi: 10.1021/acssuschemeng.2c06724 (PMC9906749; doi:10.1021/acssuschemeng.2c06724)
Supplement: Supplementary file 1 — sc2c06724_si_001.pdf [file sc2c06724_si_001.pdf]

Supplementary Information

Economic and environmental performance of an integrated CO_2 refinery

Iasonas Ioannou[a], Juan Javaloyes-Antón[b], José A. Caballero[b], Gonzalo Guillén-Gosálbez [a,*]

[a.] Institute for Chemical and Bioengineering, Department of Chemistry and Applied Biosciences, ETH Zürich, Vladimir-Prelog-Weg 1, 8093 Zürich, Switzerland.

[b.] Institute of Chemical Processes Engineering, University of Alicante, PO 99E-03080 Alicante, Spain.

*Corresponding author: gonzalo.guillen.gosalbez@chem.ethz.ch

This document contains the electronic supplementary material for the article "Economic and environmental performance of an integrated CO_2 refinery". The document is organised as follows. In Section 1, we provide the problem statement, while in Section 2 we discuss the process models which comprise the CO_2 refinery. In Section 3, the parameters and methodology used in the economic assessment are provided. Section 4 introduces the life cycle inventories (LCIs) used to assess the environmental performance of the CO_2 refinery. Finally, in Section 5, some additional figures are presented.

Total number of pages: 28
Total number of tables:14
Total number of figures: 9

Contents

Section 1: Introduction 3
Problem statement 3
Definition of scenarios and labels 3
Section 2: Process modelling 4
Methanol synthesis from CO_2 and H_2 4
Olefins synthesis from MeOH 4
Aromatics synthesis from MeOH 5
Other general descriptions 7
Sensitivity analysis on the methanol synthesis 7
Section 3: Financial analysis 10
Section 4: Data for the life cycle assessment (LCA) 12
Cooling utilities 12
Synthesis facilities 15
Methanol transport 20
Section 5: Additional results 21
Heat exchange network (HEN) 21
Supplementary figures 22
References 27

Section 1: Introduction

Problem statement

We aim to quantify the environmental and economic performance of the CO_2 refinery based on carbon capture and utilisation (CCU), which delivers a given demand for petrochemicals and LPG, and identify its operational hotspots. Therefore, we carry out an environmental and techno-economic assessment which is based on mass and energy balances and equipment dimensions obtained from process simulations. Finally, we tackle possible uncertain parameters which might influence the results of our analysis via sensitivity analysis.

Essentially, we start by simulating in Aspen HYSYS a set of technologies to design the CO_2 refinery. Namely, these technologies convert captured CO_2 and electrolytic H_2 via methanol (MeOH process) into platform chemicals and liquefied petroleum gas (LPG), via the methanol-to-olefins, MTO, and methanol-to-aromatics, MTA, process. Finally, our assessment considers the links between CO_2-based synthesis and the residual gases utilisation (RGU) from the synthesis steps which is rarely discussed, and includes decentralised and centralised facilities, allowing for an in-depth economic and environmental investigation.

Definition of scenarios and labels

The scenarios considered in the analysis and the corresponding labels are provided in **Table S1**.

Table S1. Correspondence between labels and scenarios reported in this study

Scenario	Labels	Description
Scenario 1	NIACU w/o credits	1. Decentralised manufacturing facilities (MeOH, MTO, and MTA)<br>2. Air-based combustion for the residual gases utilisation (RGU)<br>3. Heat and power co-generation and use onside<br>4. Any excess energy from RGU is wasted
Scenario 2	NIACU w credits	1. Decentralised manufacturing facilities (MeOH, MTO, and MTA)<br>2. Air-based combustion for the RGU<br>3. Heat and power co-generation and use onside<br>4. Credits for any excess energy from RGU
Scenario 3	IACU	1. Integrated manufacturing facility (CO_2 refinery)<br>2. Integrated air-based combustion for the RGU<br>3. Heat and power co-generation and use onside
Scenario 4	IOCU	1. Integrated manufacturing facility (CO_2 refinery)<br>2. Integrated oxygen-based combustion for the RGU | Allam cycle<br>3. Power generation and use onside<br>4. Recycle of pure CO_2 from the Allam cycle to the CCU synthesis

Section 2: Process modelling

Methanol synthesis from CO_2 and H_2

The CO_2-based MeOH process provides the feedstock for olefins and aromatics production. We developed an Aspen HYSYS process flowsheet based on Gonzalez-Garay et al.[1] (**Fig. S1**, top). In this process model we used two different thermodynamic packages, i.e., Peng-Robinson until stream 19 (S19) of **Fig. S1** and NRTL-Ideal afterwards. eH_2 produced via proton exchange membrane (PEM) electrolysis is compressed from 30.0 to 50.0 bar in one stage, while the CO_2 feedstock (atmospheric origin) is compressed from 1.0 to 50.0 bar in a four-stage compression train with intermediate cooling which reduces the temperature of the intermediate vapour outlets to 40 °C. The two streams are mixed with a recycled stream and preheated to the reactor's conditions. Methanol is then produced in a PFR (R-01), with a commercial Cu-ZnO-Al_2O_3 catalyst and operating at 237°C and 50.0 bar at the inlet, with an overall pressure drop of 5.0 bar.[2] The reactor's total volume amounts to 40.6 m^3, consisting of 8000 tubes of 14.2 m length, while the tubes void fraction is equal to 0.6. The temperature of the reactor's outlet is reduced to 35 °C, and the stream is fed into a flash unit (V-00). The vapour outlet of the latter unit comprises unreacted CO_2, carbon monoxide (CO), and H_2 (62.3, 22.9, and 13.7 % wt., respectively) and a small amount of methanol and water (1.1 and 0.1 % wt., respectively). This stream is recycled to the reactor after its compression (from 44.3 to 50.0 bar), purging a small fraction (0.1%) to minimise the build-up of species. Subsequently, the liquid outlet is expanded to 1.8 bar fed into a second flash unit (V-01). The liquid stream is pre-heated and enters a distillation column (T-00), where methanol attains a mass fraction of 99.9% at the liquid outlet of the partial condenser. The methanol product splits into three streams, two of them sent to the MTO and MTA synthesis facilities while the remaining fraction is sold.

Overall, 1.00 kg of methanol requires 1.43 kg of CO_2 and 1.95×10^{-1} kg of H_2 while generating 0.56 kg of wastewater as a non-valuable byproduct. Furthermore, three residual gas streams (RGU 1-3) are sent to the utilisation cycle, i.e., purge of the first flash unit (44.3 bar), the second flash unit vapour outlet (1.8 bar), and the partial condenser's vapour stream (1.0 bar), whose pressures are equalised before entering the burner of the RGU stage.

Olefins synthesis from MeOH

The Aspen HYSYS flowsheet of the MTO facility is based on some previous work (**Fig. S1**, middle).[3] In this process model, we used two different thermodynamic packages, i.e., Peng-Robinson, everywhere except for the CO_2 removal stage where we used the Acid Gas - Chemical Solvents package as discussed next. A conversion reactor (R-02) was used based on experimental yield data of a SAPO-34 catalyst,[4] where the dehydration of methanol towards olefins occurs at 1.5 bar and 450 °C. We utilise the released heat from the latter exothermic reactor in a two-stage Rankine cycle. The two-stage Rankine cycle first partly consumes the reactor's heat by evaporating water at 55 bar from 45 to 430 °C. Subsequently, the pressure is released to 2 bar via a turbine, and then the stream's temperature increases to 430 °C[5] by utilising the remaining heat of the reactor. The stream enters next to a second turbine where its pressure reduces to 0.1 bar. We, here, use a steam Ranking cycle (SRC) instead of an organic Rankine cycle (ORC) since, according to Hung et al. (1997),[5] the ORC only becomes more appealing than the SRC for a heat source with a temperature below 370°C. Finally, the steam temperature is reduced to 44 °C (1.5 °C lower than saturation temperature) and then pumped to 55 bar. Notably, the selected pathway generates a significant amount of water at the reactor's outlet, amounting to 56.1% wt. of the total. At the same time, the MTO reaction also co-produces a few chemicals with high calorific value (e.g., CO, methane [CH_4], ethane [C_2H_6], among others).

Since the purification step is based on cryogenic separation, the water needs to be removed from the reactor outlet stream to avoid the formation of hydrates. Therefore, at first, a bulk water removal step is carried out (amounting to 99.9 % of the total) based on two flash units in series (V-02 and V-03) that operate at different pressures (1.5 and 30.4 bar, respectively). The pressure build of the latter stream is achieved with a three-stage compression with intermediate cooling, which reduces the compressor's outlet stream temperature to 50, 70, and 35 ^{o}C, respectively. The two former values were chosen to reduce the stream temperature while avoiding phase change and thus, cavitation in the subsequent compression step, while the latter to maximise the bulk removal of the water by-product. On the downstream, the CO_2 present in the products' stream is captured via amine scrubbing (A-00), and the lean stream is further dehydrated with glycol via an absorption column. Subsequently, since the dry stream contains a small portion of H_2, a series of three cryogenic knockout drums (V-04, V-05 and V-06) and a pressure swing adsorption (PSA-00) unit are used for recovery and purification. The three knockout drums operate at -60, -80, and -100 ^{o}C, respectively, and all liquid streams are mixed and sent to a demethaniser distillation column (T-03) while the vapour stream is fed to the PSA unit. The knockout drums series leads to a 1.3% loss of the total ethylene inlet at the vapour outlet. Notably, operating at higher temperatures increases the ethylene loss, i.e., a -40, -60, -80 ^{o}C sequence losses 4.0%. In comparison, a lower temperatures sequence, i.e., a -80, - 100, -120 ^{o}C, could lead to a 0.3% loss but create the need for cryogenic cooling at -120 ^{o}C as trade-off. The captured H_2 can then be recycled back to the methanol synthesis process. Finally, the valuable products are recovered via a sequence of distillation columns (T-03 to T-07) with 99.9 % wt. purity.

Overall, the production of 1.00 kg of valuable aggregate products requires 2.40 kg of methanol, while 1.35 kg of wastewater is cogenerated as a non-valuable byproduct. The three RGU streams are taken from (i) the CO_2 stripper from the MEA regeneration (30.2 bar), (ii) the PSA unit (29.6 bar), and (iii) the top of the demethaniser column (29.4 bar). These streams are mixed to generate RGU 4, as illustrated in **Fig. S1** (middle).

Aromatics synthesis from MeOH

Similarly, the aromatics synthesis step was simulated based on experimental data using a conversion reactor (R-03) , and the Aspen HYSYS flowsheet is based on the literature (**Figure S1**, bottom).[6] In this process model we used the Peng-Robinson thermodynamic packages. The aromatics generation from methanol takes place at 4.0 bar and 475 °C.[7] As before, we utilise the reaction's released heat in two-stage Rankine cycles, and a high amount of coproduced water needs to be removed (52.1 % wt. at the reactor's outlet).

At first, a three-phase separation unit (V-09) operating at 4.0 bar achieves bulk water removal. The vapour stream of the separator is compressed to 6.5 bar and fed into a second three-phase flash unit (V-10). Both water separation steps attain a 99.9 % water removal. The light liquid from the first separator is pressurised and mixed with the equivalent steam of the second unit. The mixture flows to a distillation column (T-08), which separates at the bottom the BTXs group, whereas the top holds the lighter components ($C \leq 5$). The stream rich in BTXs is further fractionated into its single components (purity > 99.7 % wt.) using the direct sequence distillation arrangement (T-09, T-10 and T-11). In contrast, the vapour product of T-08 is pressurised to 10.0 bar and mixed with the vapour stream of the second separator unit, before flowing to the distillation column T-12 for recover pentane in the column's bottom stream. The vapour outlet is compressed to 18.0 bar and treaded with glycol to remove any traces of water in the absorber column A-01. Glycol is recovered in the glycol recovery column T-14. Finally, the dehydrated stream is fed in a cryogenic distillation unit (T-13). The bottom stream of the latter column containing the heavier components constitutes the LPG product.

Overall, the production of 1.00 kg of valuable aggregate products requires 2.34 kg of methanol, while 1.22 kg of wastewater is generated as a non-valuable byproduct. The vapour outlet of the LPG column primarily consists of C2 and lightweight components (RGU 5, 17.0 bar), and is sent to the RGU cycle.

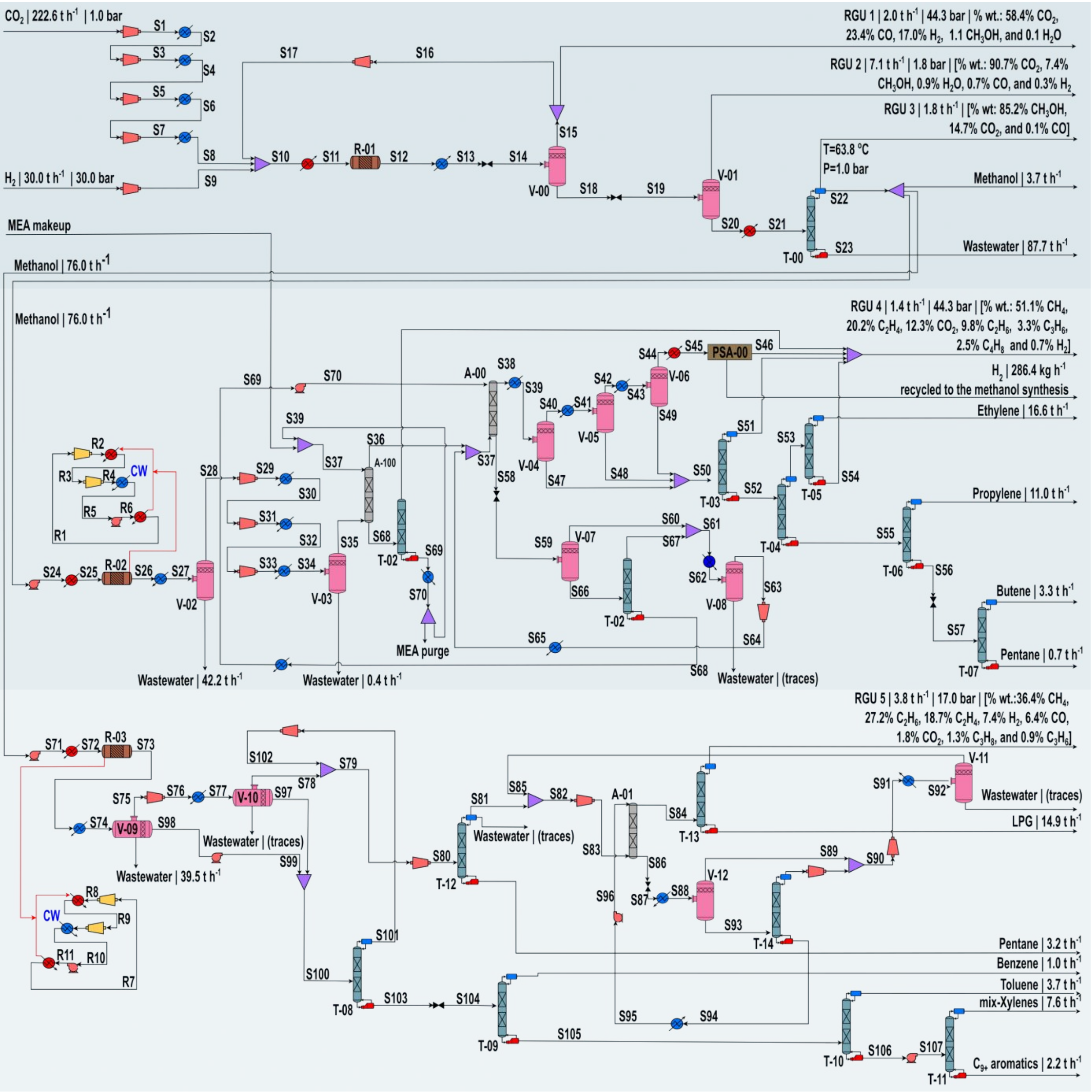

Figure S1: Process flow diagram of the CO_2 refinery: CO_2 to methanol process (top), methanol-to-olefins process (middle), and methanol-to-aromatics (MTA) process (bottom).

Other general descriptions

We highlight first some general descriptions of the modelling stages:

- An adiabatic efficiency of 80% characterises all compressors and turbines discussed in this section.
- In multi-stage compression steps, the pressure ratio between outlet and inlet pressure was limited within the range of 2- and 5-fold, i.e., $2 \leq P_{out}/P_{in} \leq 5$, and being the same for all the compressors within the same pressure conditioning stage.

Sensitivity analysis on the methanol synthesis

The conditions of the methanol synthesis process were the outcome of two sensitivity analyses for the main process parameters and represented a design with the minimum energy requirements and deviations from the ideal stoichiometric representation. Notably, the overall mass balance deviates slightly from a stoichiometric reaction, and thus, it leads to the generation of a small amount of residual gases.

The pressure and temperature at the reactor's inlet were selected through the first sensitivity analysis (see **Fig. S2**). The inflow of CO_2 and H_2 feedstock was the same as in **Fig. S1**. Notably, the pressure and temperature influence the reactants' recycling and purging streams. We, here, only considered the intervals within which the kinetic model was validated (15-50 bar and 180-280 ^oC, respectively)[1]. Since the reaction is exothermic, we should also ensure that the reactor's outlet stream becomes at most 280 ^oC. We observe that even though the amount of CO_2 and H_2 inlet was kept the same, changing the conditions in the reactor led to the production of a different methanol capacity. Thus, the per kg methanol values vary in **Fig. S2**. The best conditions for producing methanol are a high pressure and temperature at the reactor's inlet (i.e., 237 ^oC and 50 bar, leading to a 280 ^oC and 45 bar at the outlet). This occurs since a higher pressure in the reactor will lead to higher conversion and, thus, higher heat generation, which means a higher temperature at the outlet. The latter could allow the exchange of heat with the reboiler of the column that purifies methanol, considering a ΔT_{min}, and a lower recycle flow, which in turn lowers the electricity for recompression.

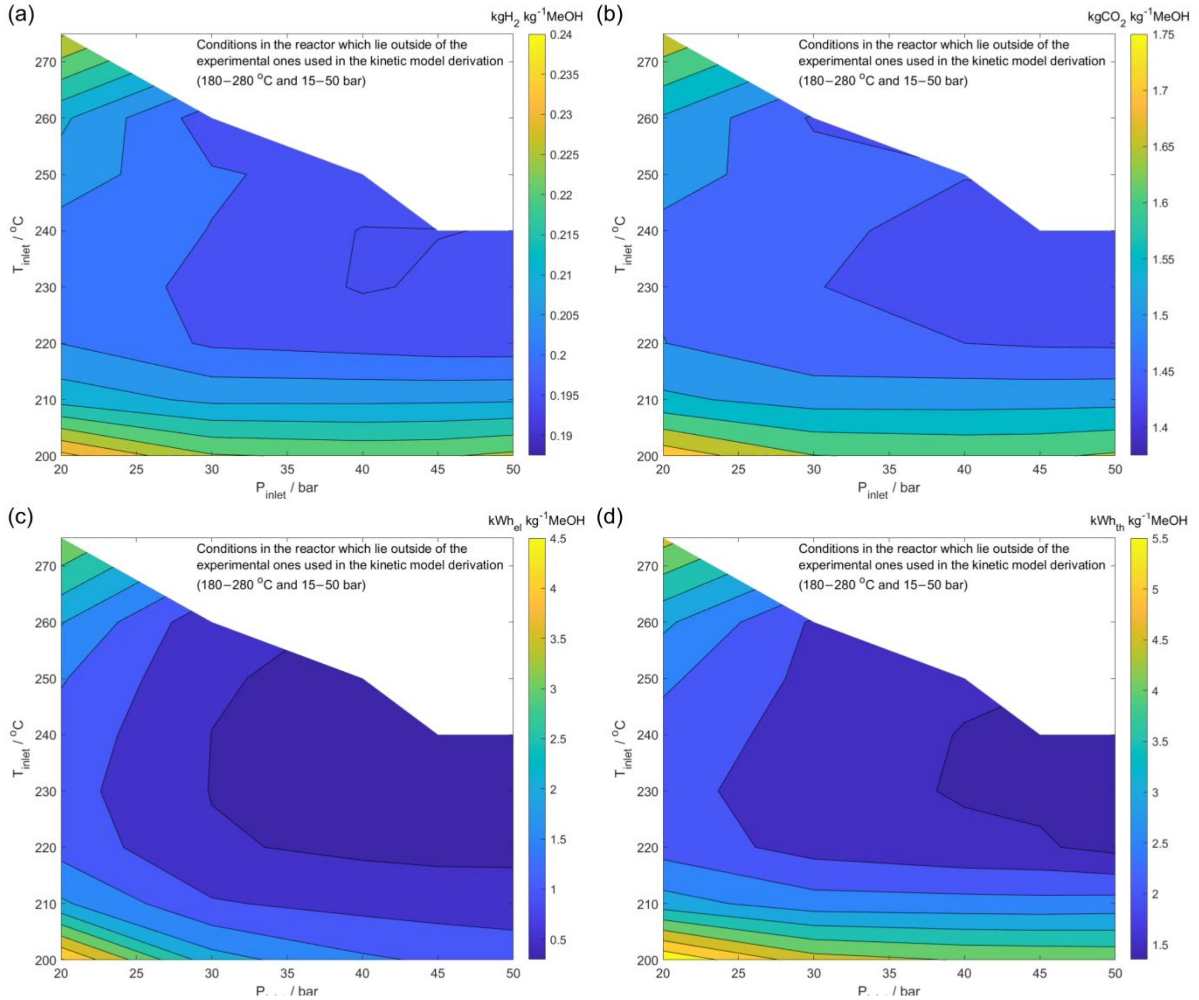

Figure S2: Sensitivity analysis of the methanol synthesis, where we vary the temperature and pressure for the reactor inlet and illustrate the amount of (a) hydrogen, (b) carbon dioxide, (c) electricity, and (d) heat consumed when producing 1 kg of methanol.

Regarding the inlet flows, one can vary the amount of CO_2 and H_2 feedstock, as illustrated in **Fig. S3,** while fixing the reactor conditions based on the trend mentioned above. We also consider a model with the ideal stoichiometric reaction counterpart for comparative purposes. We observe that the base case (which uses the reaction kinetic) show a slight deviation for the mass transformation relative to the stoichiometric counterpart. The origin of this mass deviation is the conversion per pass, the purification steps, which led to the generation of a small quantity of undesired residual gases. Furthermore, the recycle stream conditioning, i.e., recompression and preheating, leads to significantly higher energy requirements than the ideal counterpart. Besides, the stoichiometric counterpart does not require either preheating or electricity for the recompression of the recycle stream. Therefore, the column's reboiler of the stoichiometric model does not require additional heating due to the excess amount generated in the reactor. All in all, reducing (or increasing) the gap between the amount CO_2 and H_2 feedstock of the base case leads to an increase in energy requirements, which aided on the selection of the CO_2 and H_2 mass flow at the inlet.

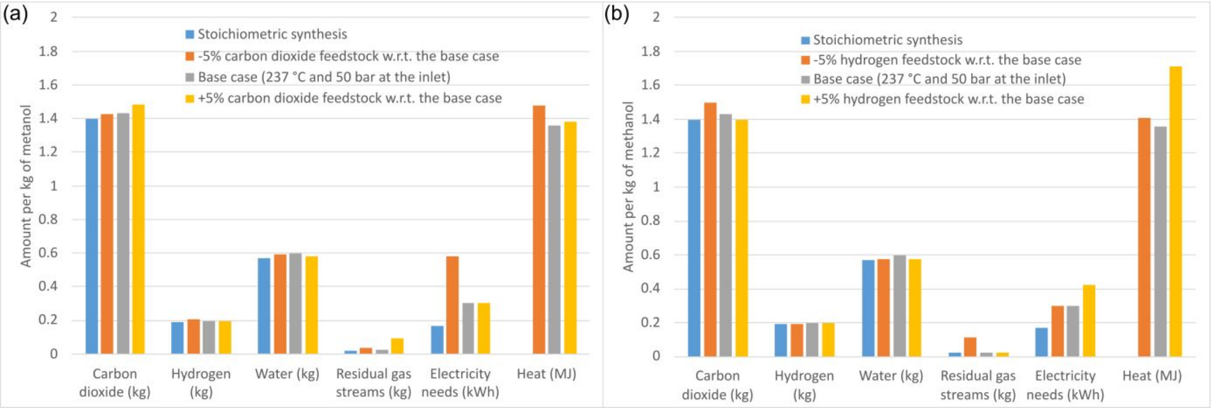

Figure S3: Sensitivity analysis of the methanol synthesis, where we vary the mass flow of the (a) CO_2 (left) and (b) H_2 (right) feedstock and illustrate the amount of carbon dioxide, hydrogen, water, residual gasses, electricity, and heat consumed or generated when producing 1 kg of methanol.

Section 3: Financial analysis

We briefly discuss the methodological approach used for the financial analysis, which follows the standard procedure for preliminary estimates. Namely, the methodological steps, correlations, and factors used are available in Towler and Sinnot (Chapters 7-9).[8] The process simulator provides the equipment units' sizes, the material and energy flows needed in estimating the revenues, capital expenditures, and finally, the variable and fixed operational cost (VOC and FOC, respectively).

Based on correlations for standard plant equipment and data from the developed flowsheets, we estimate the purchase cost for most of the designed units. We further extract from the literature cost correlations of specialised units, e.g., electrolyser, DAC, MeOH, MTO, MTA reactors, gas turbine and pressure swing adsorption.[1,9–11] Notably, we project the calculated costs to 2019 using the CEPCI index. Using typical factors, the purchased cost can then be translated into total fixed capital investment (TFCI). Namely, these factors consider inside battery limits investment (ISBL), outside battery limits investment (OSBL), engineering and construction costs, and contingency charges. Finally, the cost for purchasing and operating the methanol transportation units, i.e., tanker trucks, were based on typical methodologies available in the literature.[12]

We list in **Table S2** the cost parameters used in the economic assessment of the CO_2 refinery and for the methanol transportation calculations. Furthermore, for the FOC, we assume eight operators per shift with a salary of 40,000 $ y^{-1}, and a total of 3 shifts, to estimate the operating labour. An additional 25% of the operating labour is considered for supervision, and an extra 40% of their summation is assumed to include the direct salary overhead. Finally, we also have contributions from the (i) maintenance, (ii) property taxes and insurance, and (iii) rent of land, which is estimated as a percentage of the ISBL and OSBL.

The key economic indicator of this study is the net present value (NPV) and calculated based on the following parameters: (i) 30 y plant lifetime, (ii) 8000 h y^{-1} of operation, (iii) a 7 % interest rate, (iv) a 30 % federal income tax, and finally, 7 y MACRS depreciation charges. Here, we assume a 3-year construction period in which a share of the TFCI is charged at each period. Namely, in the (i) first year: 30 % for engineering and long lead-time items purchase, (ii) second year: 50 % for procurement and construction of the plant, and finally, (iii) third year: 20 % for the remaining construction. In the third year, the plant is assumed to deliver 30 % of the designed capacity, and thus, the working capital and the respective VOC and FOC expenses are also considered. In the final year of the plant's life, the working capital is recovered.

Table S2: Cost parameters used in the VOC and methanol transportation costs calculations

	Units	Price	Source
Electricity from the grid	$ MWh^{-1}	70.0	[13,14]
eH_2	$ t^{-1}	3000	[15]
CO_2 from DAC	$ t^{-1}	90.0	[9]
Cooling water	$ kW^{-1} y^{-1}	10.2	[16]
Refrigerated water	$ kW^{-1} y^{-1}	127.6	[16]
Wastewater treatment	$ m^{-3}	1.5	[1]
Low pressure steam (LPS)	$ GJ^{-1}	12.1	[16]
Medium pressure steam (MPS)	$ GJ^{-1}	13.5	[16]
High pressure steam (HPS)	$ GJ^{-1}	16.2	[16]
Hot oil*	$ GJ^{-1}	17.8	-
Tanker average speed	km h^{-1}	105	-
Tanker capacity	kg $trip^{-1}$	24000	[12]
Tanker availability	h d^{-1}	18	[12]
Tanker capital cost	$	100000	[12]
Driver wage	$ h^{-1}	21.28	[12]
Tanker fuel economy	km L^{-1}	6.98	[12]
Tanker fuel price	$ L^{-1}	0.78	[12]
Tanker general costs	$ d^{-1}	8.22	[12]
Load-unload time	h $trip^{-1}$	6.00	[12]
Tanker maintenance	$ km^{-1}	0.097	[12]

* Assumed 10 % higher than HPS due to the lack of data.

Section 4: Data for the life cycle assessment (LCA)

Tables S3 through **S13** display the life cycle inventories for the subsystems in our study used for the environmental evaluation of the CO_2 refinery, the system boundaries of which are depicted in **Figure S4**.

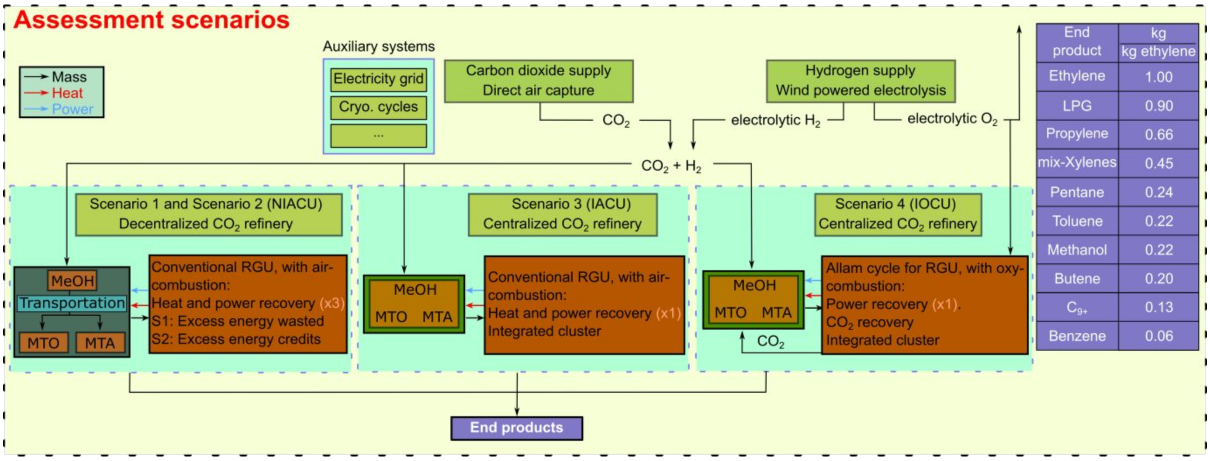

Figure S4: System boundaries of the analysed scenarios used in the LCA study.

Cooling utilities

We estimate the cooling water needs from the heat exchange network (HEN) analysis, being water heated up from 20 to 35 ºC, to satisfy the requirements of the facilities. Subsequently, the water temperature is reduced in cooling towers, where the water partly evaporates, while the rest is re-used with additional make-up to satisfy the cooling. We aimed to follow similar assumptions as in the ecoinvent database,16 from which we obtained the inventories for the raw materials, utilities, and benchmarks. Therefore, we modelled the evaporation loses as a percentage of the cooling water flow, using as a reference process the methanol plant production activity of the ecoinvent database,[17] where it is assumed that a once-through cooling system is used in 50% of all cases and a recirculating system in the remaining 50%. Finally, we introduced an electricity input representing the requirements for pumping water from 1 to 2.5 bar at 35 ºC.

Table S3: Inventory of elementary flows per MJ of cooling water produced.

Process	Material/Energy flow		Amount
Cooling water at 20 – 35 °C	Inputs:		
	Electricity*	kWh	5.53×10^{-4}
	Water, cooling, unspecified natural origin	kg	15.94
	Output, products:		
	Cooling water	MJ	1.00
	Outputs, emissions/wastes:		
	Water to air⁺	kg	6.18
	Water to water	kg	9.76

* Electricity consumption for recirculating the cooling water.
+ Based on the ecoinvent database assumption.[17]

Table S4: Process simulation results (mass and energy balances) of a 1-stage cycle for cryogenic cooling at -25 °C per MJ of cooling produced.

Process	Material/Energy flow		Amount
1-stage cryogenic cycle at -25 °C	Inputs:		
	Electricity	kWh	0.14
	Cooling water	MJ	1.52
	Ammonia*	kg	-
	Output, products:		
	Cooling with refrigerant at -25 °C	MJ	1.00
	Outputs, emissions/wastes:		
	Ammonia*	kg	-

* Assuming no leakage, and thus, no make-up of the working fluid. The amount of ammonia circulating in the cooling cycle equals 0.99 kg MJ^{-1}.

Table S5: Process simulation results (mass and energy balances) of a 2-stage cycle for cryogenic cooling at -75 °C per MJ of cooling produced.

Process	Material/Energy flow		Amount
2-stage cryogenic cycle at -75 °C	Inputs:		
	Electricity	kWh	0.42
	Cooling water	MJ	2.51
	Ethylene*	kg	-
	Propylene*	kg	-
	Output, products:		
	Cooling with refrigerant at -75 °C	MJ	1.00
	Outputs, emissions/wastes:		
	Ethylene*	kg	-
	Propylene*	kg	-

* Assuming no leakage, and thus, no make-up of the working fluid. The amount of ethylene and propylene circulating in the cooling cycle equals 3.75 and 7.14 kg MJ^{-1}, respectively.

Table S6: Process simulation results (mass and energy balances) of a 2-stage cycle for cryogenic cooling at -100 °C per MJ of cooling produced.

Process	Material/Energy flow		Amount
2-stage cryogenic cycle at -100 °C	Inputs:		
	Electricity	kWh	0.77
	Cooling water	MJ	3.79
	Ethylene*	kg	-
	Propylene*	kg	-
	Output, products:		
	Cooling with refrigerant at -100 °C	MJ	1.00
	Outputs, emissions/wastes:		
	Ethylene*	kg	-
	Propylene*	kg	-

* Assuming no leakage, and thus, no make-up of the working fluid. The amount of ethylene and propylene circulating in the cooling cycle equals 4.11 and 10.16 kg MJ^{-1}, respectively.

Table S7: Process simulation results (mass and energy balances) of the cryogenic cooling at -125 °C per MJ of cooling produced.

Process	Material/Energy flow		Amount
3-stage cryogenic cycle at -125 °C	Inputs:		
	Electricity	kWh	0.83
	Cooling water	MJ	3.91
	Methane*	kg	-
	Ethylene*	kg	-
	Propylene*	kg	-
	Output, products:		
	Cooling with refrigerant at -125 °C	MJ	1.00
	Outputs, emissions/wastes:		
	Methane*	kg	-
	Ethylene*	kg	-
	Propylene*	kg	-

* Assuming no leakage, and thus, no make-up of the working fluid. The amount of methane, ethylene and propylene circulating in the cooling cycle is equal to 3.38, 4.38 and 10.48 kg MJ^{-1}, respectively.

Synthesis facilities

Table S8: Process simulation results (mass and energy balances) of the decentralised CO_2-to-methanol (MeOH) process of Scenario 1 and 2, per kg of methanol produced.

Process	Material/Energy flow		Amount
NIACU - MeOH	Inputs:		
	Air	kg	0.07
	Carbon dioxide	kg	1.43
	Electrolytic hydrogen	kg	1.95×10^{-1}
	Methanol catalyst	kg	2.78×10^{-5}
	Electricity	kWh	0.30
	Low pressure steam	MJ	1.14
	Cooling water	MJ	6.35
	Output, products:		
	Methanol	kg	1.00
	Outputs, emissions/wastes:		
	CO_2 to air	kg	0.08
	Water to air	kg	0.01
	Wastewater treatment	m^3	$6.10\ \times10^{-4}$

Table S9: Process simulation results (mass and energy balances) of the decentralised methanol-to-olefins (MTO) process of Scenario 1 and 2, per kg of ethylene produced.

Process	Material/Energy flow		Amount
NIACU -MTO	Inputs:		
	Air	kg	2.77
	Methanol with transport (see **Table S13**)	kg	4.58
	MEA make-up	kg	3.78×10^{-5}
	Low pressure steam	MJ	0.83
	Cooling with refrigerant at -25 °C	MJ	0.43
	Cooling with refrigerant at -75 °C	MJ	1.51
	Cooling with refrigerant at -100 °C	MJ	0.03
	Cooling with refrigerant at -125 °C	MJ	0.03
	Cooling water	MJ	5.52
	Refrigerated water	MJ	0.00
	Output, products:		
	Ethylene	kg	1.00
	Propylene	kg	0.66
	Butene	kg	0.20
	Pentane	kg	0.05
	Hydrogen	kg	0.02
	Electricity	kWh	0.23
	Outputs, emissions/wastes:		
	CO_2 to air	kg	0.22
	Water to air	kg	0.14
	MEA to water	kg	3.78×10^{-5}
	Wastewater treatment	m^3	2.59×10^{-3}

Table S10: Process simulation results (mass and energy balances) of the decentralised methanol-to-aromatics (MTA) process of Scenario 1 and 2, per kg of LPG produced.

Process	Material/Energy flow		Amount
NIACU -MTA	Inputs:		
	Air	kg	9.79
	Methanol with transport (see **Table S13**)	kg	5.11
	Cooling with refrigerant at -75 °C	MJ	0.63
	Cooling water	MJ	9.19
	Refrigerated water	MJ	0.32
	Output, products:		
	Liquefied petroleum gas	kg	1.00
	Xylenes	kg	0.51
	Toluene	kg	0.25
	Benzene	kg	0.07
	Pentane	kg	0.21
	C_{9+} (burden free product)	kg	0.14
	Electricity	kWh	1.01
	LPS **(only in the scenario with credits)**	MJ	6.35
	Outputs, emissions/wastes:		
	CO_2 to air	kg	0.66
	Water to air	kg	0.46
	Wastewater treatment	m^3	2.77 x10^{-3}

Table S11: Process simulation results (mass and energy balances) of the integrated CO_2 refinery of Scenario 3, per kg of ethylene produced.

Process	Material/Energy flow		Amount
IACU	Inputs:		
	Air	kg	15.68
	Carbon dioxide	kg	13.40
	Electrolytic hydrogen	kg	1.81
	MEA make-up	kg	$3.78x10^{-5}$
	Electricity	kWh	1.22
	Low pressure steam	MJ	3.99
	Cooling with refrigerant at -25 °C	MJ	0.43
	Cooling with refrigerant at -75 °C	MJ	2.08
	Cooling with refrigerant at -100 °C	MJ	0.03
	Cooling with refrigerant at -125 °C	MJ	0.03
	Cooling water	MJ	74.04
	Refrigerated water	MJ	0.29
	Output, products:		
	Ethylene	kg	1.00
	Liquefied petroleum gas	kg	0.90
	Propylene	kg	0.66
	Xylene	kg	0.45
	Pentane	kg	0.24
	Toluene	kg	0.22
	Butene	kg	0.20
	Methanol	kg	0.22
	Benzene	kg	0.06
	C_{9+} (burden free product)	kg	0.13
	Outputs, emissions/wastes:		
	CO_2 to air	kg	1.45
	Water to air	kg	0.76
	MEA to water	kg	$3.78x10^{-5}$
	Wastewater treatment	m^3	$1.09 x10^{-2}$

Table S12: Process simulation results (mass and energy balances) of the integrated CO_2 refinery of Scenario 4, per kg of ethylene produced.

Process	Material/Energy flow		Amount
IOCU	Inputs:		
	Carbon dioxide	kg	11.91
	Electrolytic hydrogen	kg	1.81
	Electrolytic oxygen	kg	1.51
	MEA make-up	kg	$3.78x10^{-5}$
	Low pressure steam	MJ	17.22
	Medium pressure steam	MJ	0.40
	High pressure steam	MJ	0.88
	Hot oil	MJ	0.62
	Cooling with refrigerant at -25 °C	MJ	0.43
	Cooling with refrigerant at -75 °C	MJ	2.08
	Cooling with refrigerant at -100 °C	MJ	0.03
	Cooling with refrigerant at -125 °C	MJ	0.03
	Cooling water	MJ	83.06
	Refrigerated water	MJ	0.29
	Output, products:		
	Ethylene	kg	1.00
	Liquefied petroleum gas	kg	0.90
	Propylene	kg	0.66
	Xylene	kg	0.45
	Pentane	kg	0.24
	Toluene	kg	0.22
	Butene	kg	0.20
	Methanol	kg	0.22
	Benzene	kg	0.06
	Electricity	kWh	0.94
	C_{9+} (burden free product)	kg	0.13
	Outputs, emissions/wastes:		
	MEA to water	kg	$3.78x10^{-5}$
	Wastewater treatment	m^3	$1.16\ x10^{-2}$

Methanol transport

Table S13: Inventory of elementary flows per kg of methanol transported.

Process	Material/Energy flow		Amount
Methanol with transport	Inputs:		
	Methanol	kg	1.00
	Transport, freight, lorry, unspecified*	ton*km	2.16×10^{-1}
	Output, products:		
	Methanol with transport	kg	1.00

* Adapted from ecoinvent market for methanol[17]

Section 5: Additional results

Heat exchange network (HEN)

Heat integration is carried out using the sequential approach described in Floudas et al.[18] using a heat recovery approach temperature of 10 °C. This approach is based on solving three optimisation problems. The linear extended transshipment problem (minimum utility cost) is first solved, followed by a the mixed-integer linear transhipment problem for the minimum number of units.[19] A non-linear automatic synthesis of the optimal HEN for minimum investment cost is finally performed.

The heating targets (**Table S14**) for the designed capacity of the CO_2 refinery are 58.1, 16.6, and 13.4 MW for the MeOH, MTO, and MTA processes, respectively. Furthermore, the cooling targets are significantly higher when compared to the heating target, amounting to 274.3, 33.1, and 36.4 MW (following the same sequence as before). Finally, the pinch temperature for the MeOH, MTO and MTA processes is 119.3 – 109.3, 84.8 – 74.8, 116.1 – 106.1 °C, respectively. The heating and cooling utility needs are summarised in **Table S14**. The optimal HEN descriptions lead to the design of the conventional RGU for heat generation.

Table S14: Utility targets breakdown of the three main processes of the CO_2 refinery.

Utility type	Temp.	MeOH	MTO	MTA
Heating utilities	[°C]	[MW]	[MW]	[MW]
Hot oil	500-550	–	2.30	0.50
HPS (41 barg)	254	–	–	4.00
MPS (10 barg)	184	–	–	1.80
LPS (5 barg)	160	58.10	14.3	7.00
Cooling utilities				
Cooling water	20 – 35	274.30	23.90	32.40
Refrigerated water	5 – 15	–	–	1.30
1-stage cycle*	-25	–	1.97	–
2-stage cycle 1*	-75	–	6.98	2.60
2- stage cycle 2*	-100	–	0.16	–
3-stage cycle*	-125	–	0.14	–

* The cryogenic refrigeration is suppled based on 1-, 2-, and 3-stage cryogenic cycles simulated in Aspen HYSYS based on

Supplementary figures

In this section, we provide in **Fig. S5** some additional results on variable and fixed operational costs (VOC and FOC, respectively), while **Fig. S6** depicts the sensitivity analysis on the NPV. Moreover, **Fig. S7,** illustrates the midpoint avitivites breakdown for the base case, i.e., the CO_2 refinery operates in Germany. Finally, **Fig. S8** and **S9** depict the sensitivity of the global warming impact of the CO_2 refinery for different European locations.

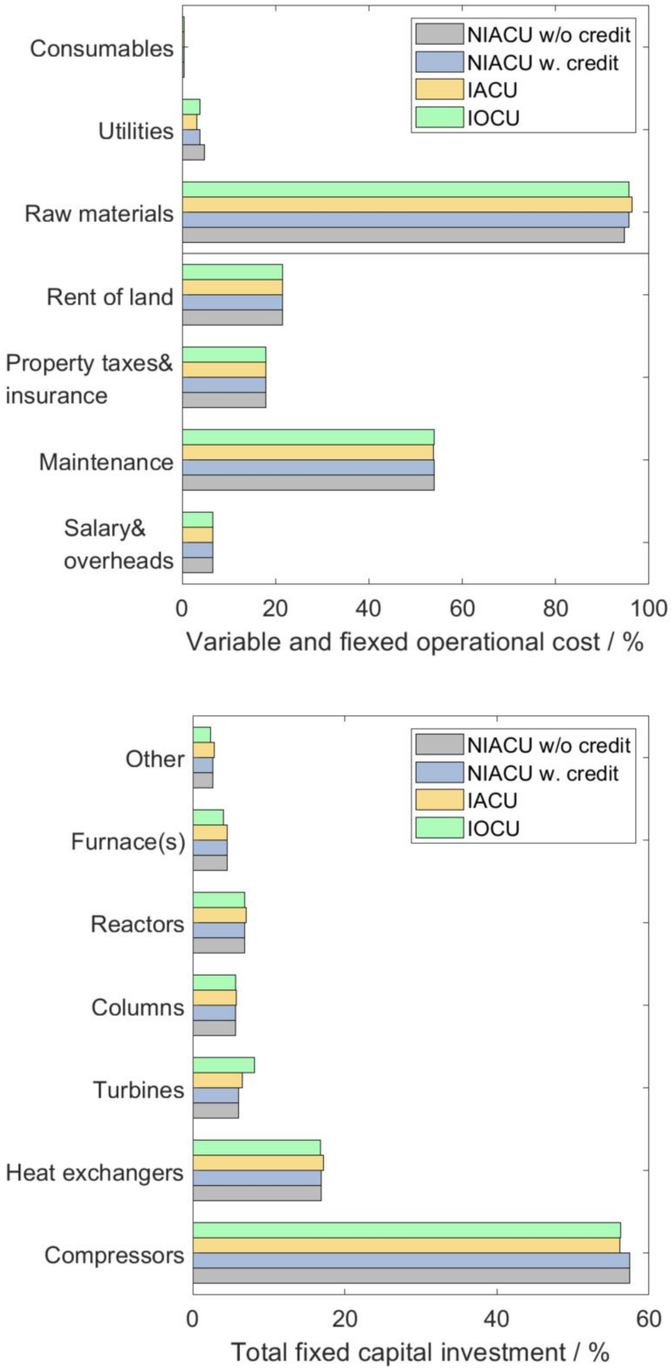

Figure S5. Breakdown of the VOC and FOC (top), and TFCI (bottom) for the four scenarios of the CO_2 refinery.

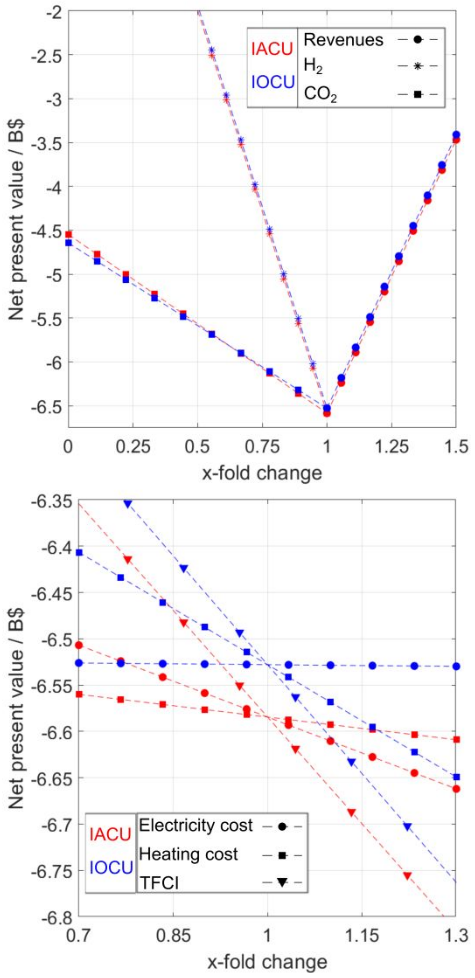

Figure S6. Net present value sensitivity on key parameters for the two centralised scenarios of the CO_2 refinery.

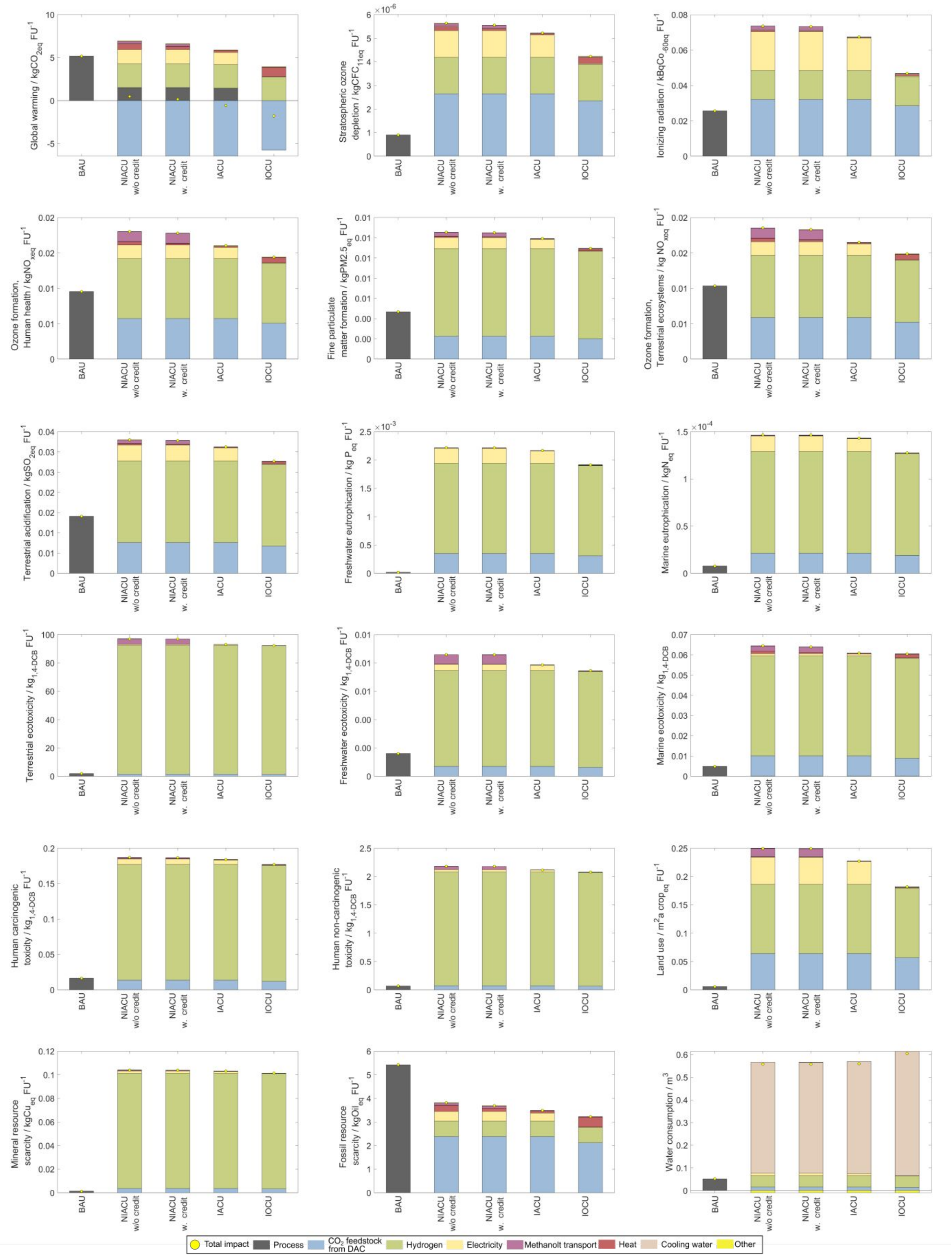

Figure S7: Environmental impact on the 18 midpoints of the ReCiPe 2016 (H) methodology.

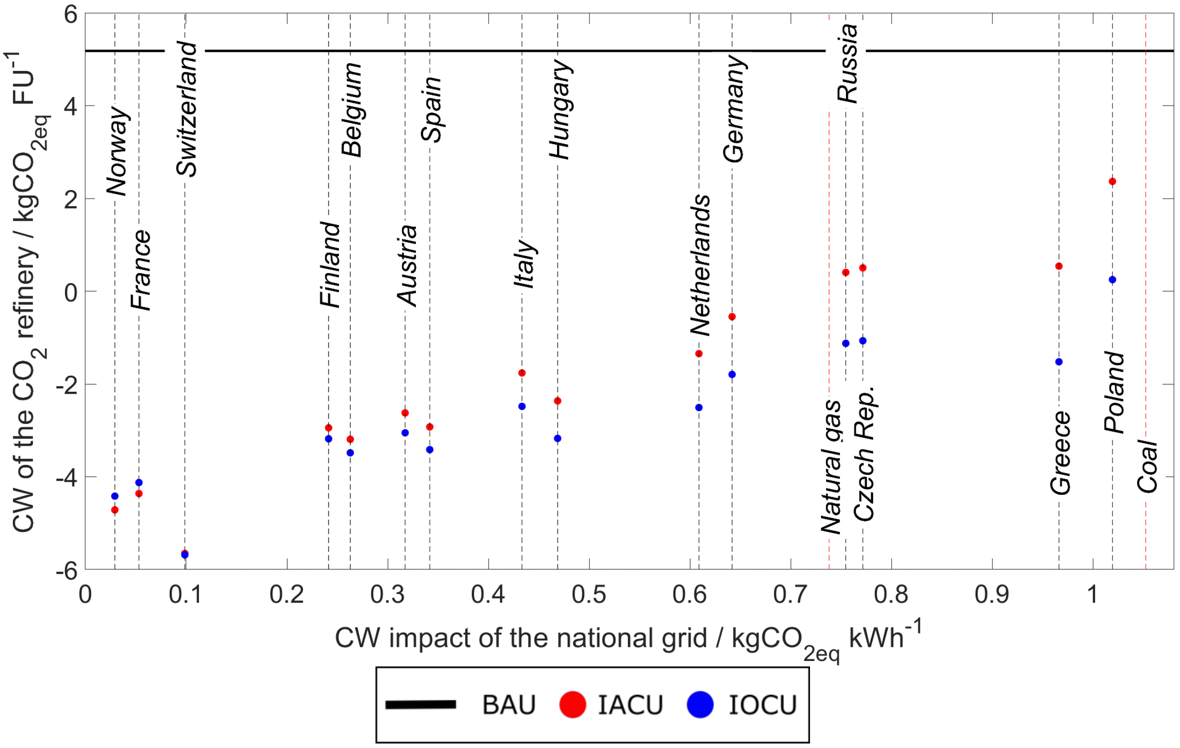

Figure S8: Environmental burden on global warming (GW) of the CO_2 refinery per FU in different regions of the world vs the global warming (GW) impact on the local power grid.

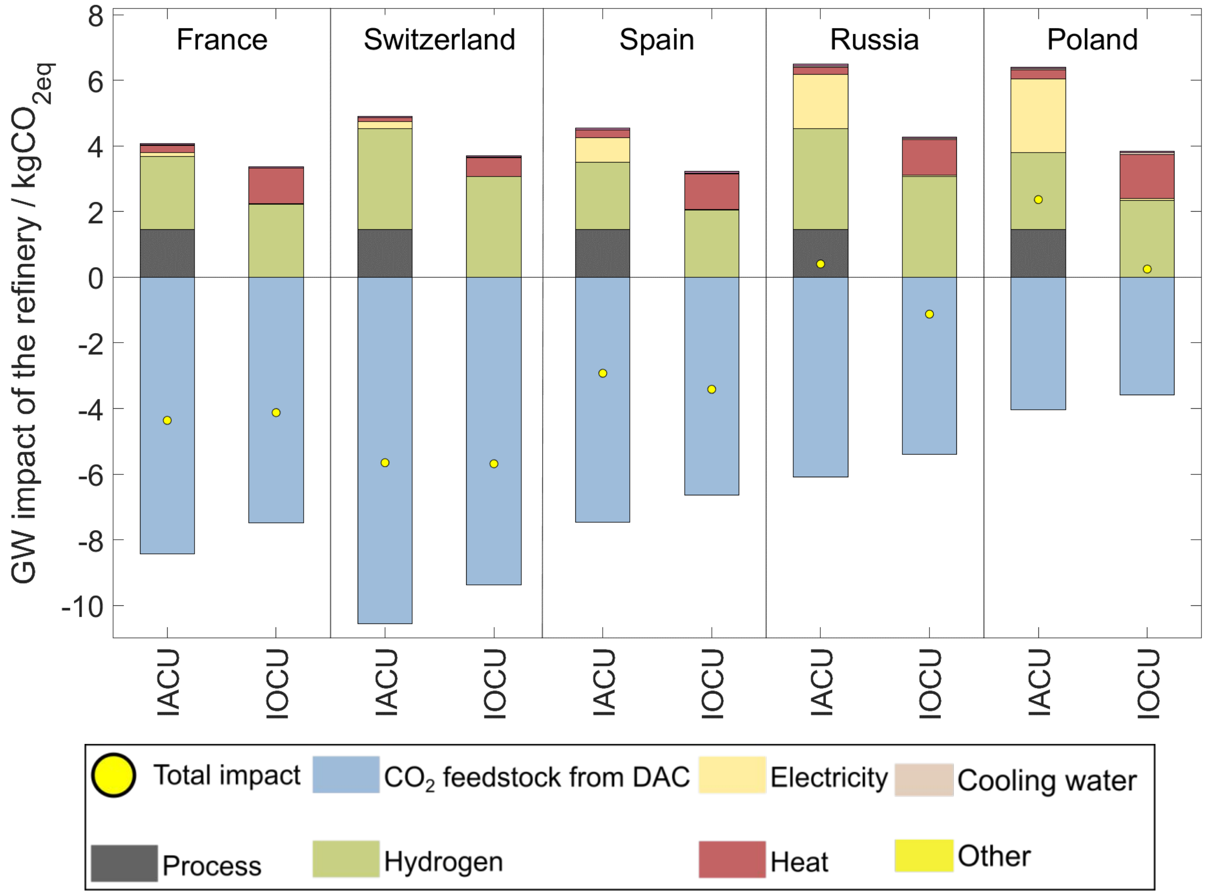

Figure S9. Breakdown of the environmental burden on global warming (GW) of the CO_2 refinery per FU in different regions of the world vs the global warming (GW) impact on the local power grid.

References

(1) González-Garay, A.; Frei, M. S.; Al-Qahtani, A.; Mondelli, C.; Guillén-Gosálbez, G.; Pérez-Ramírez, J. Plant-to-Planet Analysis of CO_2-Based Methanol Processes. *Energy Environ. Sci.* **2019**, *12* (12), 3425–3436. https://doi.org/10.1039/c9ee01673b.

(2) Martin, O.; Mondelli, C.; Cervellino, A.; Ferri, D.; Curulla-Ferré, D.; Pérez-Ramírez, J. Operando Synchrotron X-ray Powder Diffraction and Modulated-Excitation Infrared Spectroscopy Elucidate the CO_2 Promotion on a Commercial Methanol Synthesis Catalyst. *Angew. Chemie Int. Ed.* **2016**, *55* (37), 11031–11036. https://doi.org/10.1002/anie.201603204.

(3) Ioannou, I.; D'Angelo, S. C.; Martín, A. J.; Pérez-Ramírez, J.; Guillén-Gosálbez, G. Hybridization of Fossil-and CO_2-based Routes for Ethylene Production Using Renewable Energy. *ChemSusChem* **2020**, *13* (23), 6370–6380. https://doi.org/10.1002/cssc.202001312.

(4) Lee, Y.-J.; Baek, S.-C.; Jun, K.-W. Methanol Conversion on SAPO-34 Catalysts Prepared by Mixed Template Method. *Appl. Catal. A,* **2007**, *329*, 130–136. https://doi.org/10.1016/j.apcata.2007.06.034.

(5) Hung, T. C.; Shai, T. Y.; Wang, S. K. A Review of Organic Rankine Cycles (ORCs) for the Recovery of Low-Grade Waste Heat. *Energy* **1997**, *22* (7), 661–667. https://doi.org/10.1016/S0360-5442(96)00165-X.

(6) Zhang, D.; Yang, M.; Feng, X. Aromatics Production from Methanol and Pentane: Conceptual Process Design, Comparative Energy and Techno-Economic Analysis. *Comput. Chem. Eng.* **2019**, *126*, 178–188. https://doi.org/10.1016/j.compchemeng.2019.04.002.

(7) Su, C.; Qian, W.; Xie, Q.; Cui, Y.; Tang, X.; Yu, X.; Wang, T.; Huang, X.; Wei, F. Conversion of Methanol with C5–C6 Hydrocarbons into Aromatics in a Two-Stage Fluidized Bed Reactor. *Catal. Today* **2016**, *264*, 63–69. https://doi.org/10.1016/j.cattod.2015.09.022.

(8) Towler, G. P.; Sinnott, R. K. *Chemical Engineering Design : Principles, Practice, and Economics of Plant and Process Design*; Butterworth-Heinemann: Oxford, 2012.

(9) Fasihi, M.; Efimova, O.; Breyer, C. Techno-Economic Assessment of CO_2 Direct Air Capture Plants. *J. Cleaner Prod.* **2019**, *224*, 957–980. https://doi.org/10.1016/j.jclepro.2019.03.086.

(10) Niziolek, A. M.; Onel, O.; Guzman, Y. A.; Floudas, C. A. Biomass-Based Production of Benzene, Toluene, and Xylenes via Methanol: Process Synthesis and Deterministic Global Optimization. *Energy & Fuels* **2016**, *30* (6), 4970–4998. https://doi.org/10.1021/acs.energyfuels.6b00619.

(11) Onel, O.; Niziolek, A. M.; Floudas, C. A. Optimal Production of Light Olefins from Natural Gas via the Methanol Intermediate. *Ind. Eng. Chem. Res.* **2016**, *55* (11), 3043–3063. https://doi.org/doi: 10.1021/acs.iecr.5b04571.

(12) Kostin, A. M.; Guillén-Gosálbez, G.; Mele, F. D.; Bagajewicz, M. J.; Jiménez, L. A Novel Rolling Horizon Strategy for the Strategic Planning of Supply Chains. Application to the Sugar Cane Industry of Argentina. *Comput. Chem. Eng.* **2011**, *35* (11), 2540–2563. https://doi.org/10.1016/j.compchemeng.2011.04.006.

(13) IRENA (2019)*, Renewable Power Generation Costs in 2018, International Renewable Energy Agency, Abu Dhabi.*

(14) IEA, *World Energy Outlook 2019, IEA, Paris*. https://www.iea.org/reports/world-energy-outlook-2019.

(15) IEA, *Projected Costs of Generating Electricity 2020*; Paris, 2020.

(16) Turton, R.; Bailie, R. C.; Whiting, W. B.; Shaeiwitz, J. A. *Analysis, Synthesis and Design of*

Chemical Processes; Pearson Education, 2008.

(17) Wernet, G.; Bauer, C.; Steubing, B.; Reinhard, J.; Moreno-ruiz, E.; Weidema, B. The Ecoinvent Database Version 3 (Part I): Overview and Methodology. *Int. J. Life Cycle Assess.* **2016**, *3*, 1218–1230. https://doi.org/10.1007/s11367-016-1087-8.

(18) Floudas, C. A.; Ciric, A. R.; Grossmann, I. E. Automatic Synthesis of Optimum Heat Exchanger Network Configurations. *AIChE J.* **1986**, *32* (2), 276–290. https://doi.org/10.1002/aic.690320215.

(19) Papoulias, S. A.; Grossmann, I. E. A Structural Optimization Approach in Process Synthesis-II. Heat Recovery Networks. *Comput. Chem. Eng.* **1983**, *7* (6), 707–721. https://doi.org/10.1016/0098-1354(83)85023-6.

(20) Luyben, W. L. Estimating Refrigeration Costs at Cryogenic Temperatures. *Comput. Chem. Eng.* **2017**, *103*, 144–150. https://doi.org/10.1016/j.compchemeng.2017.03.013.
